# Novel Positive Regulatory Role for the SPL6 Transcription Factor in the N TIR-NB-LRR Receptor-Mediated Plant Innate Immunity

**DOI:** 10.1371/journal.ppat.1003235

**Published:** 2013-03-14

**Authors:** Meenu S. Padmanabhan, Shisong Ma, Tessa M. Burch-Smith, Kirk Czymmek, Peter Huijser, Savithramma P. Dinesh-Kumar

**Affiliations:** 1 Department of Plant Biology and The Genome Center, College of Biological Sciences, University of California, Davis, California, United States of America; 2 Department of Biochemistry, Cellular, and Molecular Biology, University of Tennessee, Knoxville, Tennessee, United States of America; 3 Department of Biological Sciences, Delaware Biotechnology Institute, University of Delaware, Newark, Delaware, United States of America; 4 Department of Comparative Development and Genetics, Max Planck Institute for Plant Breeding Research, Cologne, Germany; Massachusetts General Hospital, Harvard Medical School, United States of America

## Abstract

Following the recognition of pathogen-encoded effectors, plant TIR-NB-LRR immune receptors induce defense signaling by a largely unknown mechanism. We identify a novel and conserved role for the SQUAMOSA PROMOTER BINDING PROTEIN (SBP)-domain transcription factor SPL6 in enabling the activation of the defense transcriptome following its association with a nuclear-localized immune receptor. During an active immune response, the *Nicotiana* TIR-NB-LRR N immune receptor associates with NbSPL6 within distinct nuclear compartments. NbSPL6 is essential for the N-mediated resistance to *Tobacco mosaic virus*. Similarly, the presumed Arabidopsis ortholog AtSPL6 is required for the resistance mediated by the TIR-NB-LRR RPS4 against *Pseudomonas syringae* carrying the avrRps4 effector. Transcriptome analysis indicates that AtSPL6 positively regulates a subset of defense genes. A pathogen-activated nuclear-localized TIR-NB-LRR like N can therefore regulate defense genes through SPL6 in a mechanism analogous to the induction of MHC genes by mammalian immune receptors like CIITA and NLRC5.

## Author Summary

Pathogen infection causes significant economic loss of crops worldwide. To fend off pathogens, plants use the Nucleotide-Binding domain and Leucine Rich Repeat (NB-LRR) class of immune receptors. Although we have some insight into how plant NB-LRRs recognizes pathogens, we know little about NB-LRR spatial distribution and dynamics during the immune response. Some plant NB-LRRs are present in the nuclear compartment of the cell suggesting that they may directly control defense gene expression. The tobacco N immune receptor that provides immunity against *Tobacco mosaic virus* (TMV) infection is present in the nucleus and associates with the SQUAMOSA PROMOTER BINDING PROTEIN-LIKE 6 (SPL6) transcription factor. This association is detected only when the TMV effector, p50, is present in the cell. This suggests that N associates with SPL6 only during an active defense response. SPL6 function is required for defense against TMV. SPL6 from Arabidopsis functions in resistance against the bacterial pathogen *Pseudomonas syringae* expressing the AvrRps4 effector and positively modulates defense gene expression. These findings define a novel conserved function for SPL6 transcription factor from different plants species in defense against pathogens. This is the first evidence for the function of SPL-type transcription factors in defense.

## Introduction

Plants employ the Nucleotide Binding-Leucine Rich Repeat (NB-LRR) family of intracellular receptors to detect pathogens and initiate defense signaling [1], [2]. NB-LRRs have structural similarity with the mammalian NOD-like receptors (NLRs), but unlike NLRs that recognize conserved Pathogen Associated Molecular Patterns (PAMPs), each plant NB-LRR recognizes a unique pathogen-encoded effector protein. NB-LRR association with an effector and subsequent receptor activation leads to a number of cellular responses that includes massive transcriptional reprogramming [3]. Ultimately, these responses often culminate in a specialized form of programmed cell death (PCD) - the hypersensitive response (HR) that restricts pathogen to the infection site thereby protecting the rest of the plant from disease [4].

Several plant NB-LRRs have been shown to localize to the nucleus, which suggests that they may participate in defense transcriptome reprogramming (reviewed in [5]). Barley CC-NB-LRR MLA10 associates with HvWRKY1 and HvWRKY2 transcriptional repressors in the presence of the AVR_A10_ effector [6]. Arabidopsis TIR-NB-LRR SNC1 associates with the transcriptional repressor TOPLESS-RELATED 1 (TPR1) to negatively regulate expression of known defense suppressors [7]. Arabidopsis RRS1-R is an atypical immune receptor that has the TIR-NB-LRR domains fused to a C-terminal WRKY domain which is characteristic of WRKY-type plant transcription factors [8]. RRS1-R recognizes the Pop2 effector from *Ralstonia solanacearum* and was observed in the nucleus only during an active immune response [9]. Interestingly, mammalian NLR proteins CIITA and NLRC5 are present in the nucleus and interact with transcription factors to promote the transcription of major histocompatibility complex (MHC) class II and class I genes [10], [11]. However, plant NB-LRR interaction with a positive regulator of defense gene transcription has not been described.

The *Nicotiana* TIR-NB-LRR immune receptor N, provides immunity against all strains of *Tobacco mosaic virus* (TMV) [12] except the TMV-Ob strain [13]. N specifically recognizes the 50 kD helicase domain (herein referred to as p50-U1) within the 126 kD replicase of TMV-U1 [14], [15]. Recognition of p50-U1 is specific because N-mediated responses are not activated by p50 from the TMV-Ob replicase (herein referred to as p50-Ob). N recognizes p50-U1 indirectly by detecting a change in the localization of an intermediary interacting protein - the chloroplast-localized N Receptor Interacting Protein 1 (NRIP1) [16]. While viral effector recognition occurs in the cytoplasm, the nuclear localization of N is required for defense signaling [17].

Here we show that the N immune receptor associates with the SQUAMOSA PROMOTER BINDING PROTEIN-LIKE 6 (SPL6) transcription factor during an active immune response. SPLs are defined by the presence of the conserved DNA-binding SQUAMOSA PROMOTER BINDING PROTEIN (SBP) domain [18]. SBP-domain containing proteins are ubiquitously found in the plant kingdom, from algae to higher plants. A subset of *SPL*s are regulated by the microRNA (miR) 156/157 [19]–[21]. Many of the characterized SPLs have been found to regulate flowering time, leaf development, transition from juvenile to adult phase, and pollen development (reviewed in [21], [22]). SPLs role in immunity however, has not been described.

We provide genetic and molecular evidence that the SPL6 transcription factor is required for N-mediated resistance to TMV. N and SPL6 associate *in planta* only in the presence of p50-U1 effector from the defense eliciting TMV-U1 strain and not in the presence of non-eliciting p50-Ob. These results indicate that only p50-U1-activated N associates with SPL6. Consistent with these observations, a mutation in the P-loop within the NB domain of N that prevents its activation also abolishes N's association with SPL6. We show that Arabidopsis SPL6 is required for the function of TIR-NB-LRR RPS4 but not for CC-NB-LRRs RPS2 and RPM1. Using Arabidopsis whole genome microarray analysis, we show that SPL6 can potentially positively regulate RPS4-mediated defense gene expression. These results point to a conserved role for SPL6 in TIR-NB-LRR-mediated immunity. Our findings support a model in which an effector-activated immune receptor associates with a positive transcriptional regulator like SPL6 to induce successful innate immune responses.

## Results

### N immune receptor interacts with the SPL6 transcription factor

**Figure 1:**
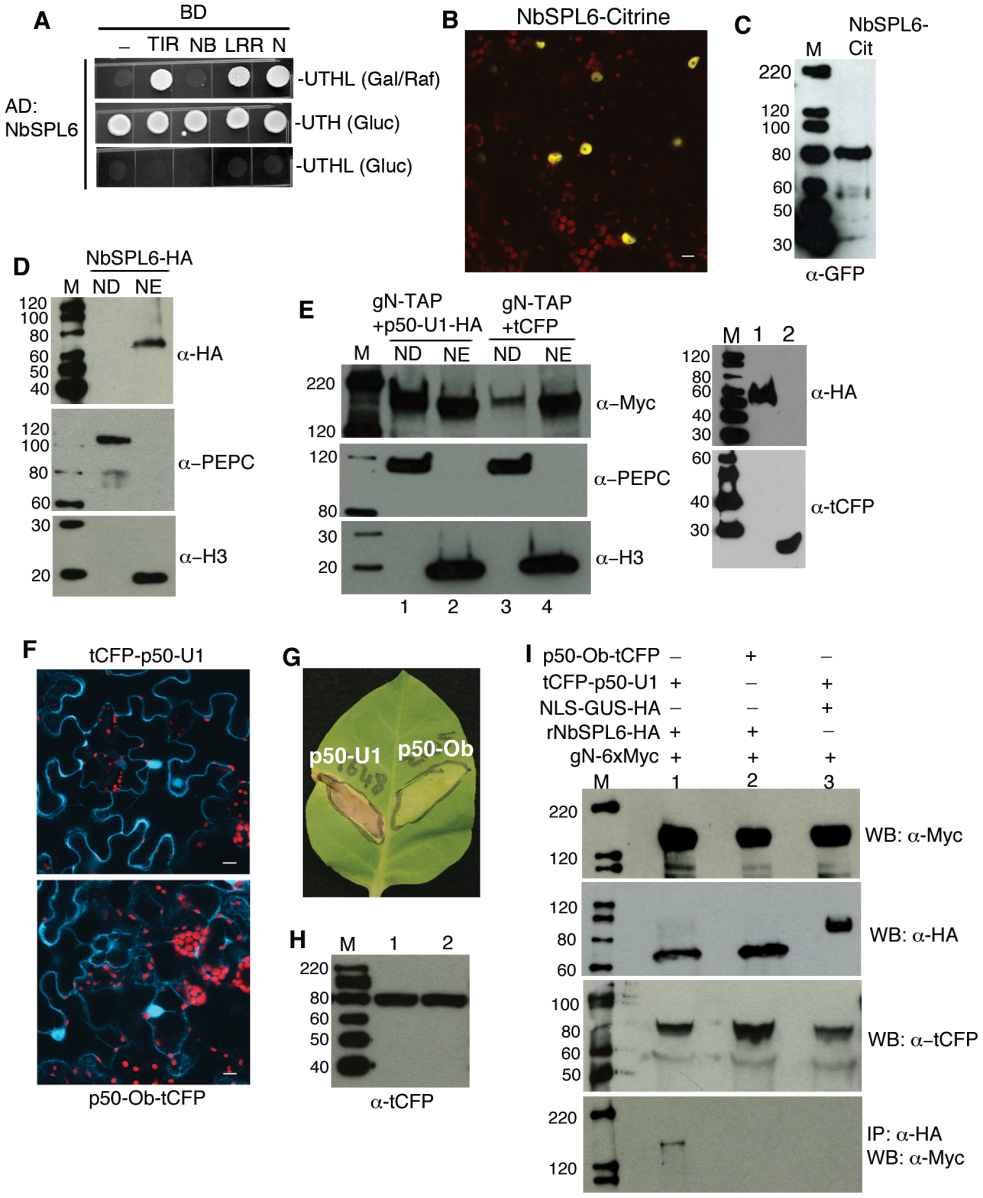
The N immune receptor associates with the NbSPL6 transcription factor during an active immune response. **A.** NbSPL6 interacts with the full-length N, TIR domain and LRR domain in a yeast two-hybrid assay as determined by growth of yeast on media lacking leucine (top panel). **B.** *N. benthamiana* cells transiently expressing NbSPL6-citrine show nuclear localization. Scale bar = 10 µm. The red structures are chloroplasts. **C.** Western blot analysis of nuclei-enriched protein fraction from tissue expressing NbSPL6-citrine followed by detection using anti-GFP antibody. M indicates marker. Protein sizes marked on the left are in kD. **D.** Western blot analysis of nuclear depleted (ND) and nuclear enriched (NE) protein fractions from NbSPL6-HA expressing tissue. NbSPL6-HA was detected only in the NE fraction (upper panel). PEPC was used as a cytoplasmic marker (middle panel) and Histone 3 (H3) was used as a nuclear marker (bottom panel). The NE fraction is approximately 16 fold concentrated over the ND fraction. M indicates marker. Protein sizes marked on the left are in kD. **E.** Cellular fractionation of tissue expressing gN-TAP with p50-U1-HA or tCFP. Left panels: gN-TAP in the presence of p50-U1-HA (lanes 1 and 2) or tCFP (lanes 3 and 4) was detected in both ND (lanes 1 and 3) and NE (lanes 2 and 4) fractions (upper panel); PEPC was used as a cytoplasmic marker (middle panel) and Histone 3 (H3) was used as a nuclear marker (bottom panel). Right panels: tissue co-expressing gN-TAP+p50-U1-HA showing presence of p50-U1-HA (lane 1, upper panel) and tissue co-expressing gN-TAP+tCFP showing presence of tCFP in the total protein extracts. M indicates marker. Protein sizes marked on the left are in kD. **F.** *N. benthamiana* cells transiently expressing tCFP-p50-U1 (upper panel) and p50-Ob-tCFP (bottom panel) show cytoplasmic and nuclear localization of the tagged proteins. Scale bar = 10 µm. The red structures are chloroplasts. **G.** Leaf sectors on *N. tabacum* cv. Glurk (NN) showing HR-PCD after transient expression of tCFP-p50-U1 (left) but not after infiltration with p50-Ob-tCFP (right). Leaf images were taken 3 days post infiltration. **H.** Western blot analysis of samples described in F showing tCFP-p50-U1 (lane1) and p50-Ob-tCFP (lane 2). M indicates marker. Protein sizes marked on the left are in kD. **I.** Co-immunoprecipitation of gN-6xMyc with rNbSPL6-HA in the presence of the N eliciting p50-U1 or non-eliciting p50-Ob. Western blot analysis confirmed expression of the input proteins: gN-6xMyc (panel 1), rNbSPL6-HA (panel 2, lanes 1 and 2), NLS-GUS-HA (panel 2, lane 3), tCFP-p50-U1 (panel 3, lanes 1 and 3), and p50-Ob-tCFP (panel 3, lane 2). Due to high expression, NLS-GUS-HA (panel 2) was adjusted to 1/50^th^ the volume loaded in lanes 1 and 2. gN-6Myc co-immunoprecipitated with rNbSPL6 only in the tissue expressing tCFP-p50-U1 (panel 4, lane 1) but not in the tissue expressing p50-Ob-tCFP (panel 4, lane 2). gN-6xMyc did not co-immunoprecipitate with NLS-GUS-HA in the presence of tCFP-p50-U1 (panel 4, lane 3). M indicates marker. Protein sizes marked on the left are in kD.

We identified 14 clones representing an SPL family member that interacted with N in a yeast two-hybrid screen. Full-length amino acid sequence of the *N. benthamiana* SPL that interacts with N indicated that it is most similar to Arabidopsis SPL6 (AtSPL6 - At1g69170). The two proteins share 83% identity within the SBP domain and 35% identity and 48% similarity within the full protein (Figure S1). Further yeast two-hybrid analysis indicated that NbSPL6 interacted with the full-length N or its TIR and LRR domains (Figure 1A).

### N associates with NbSPL6 *in planta* only in the presence of the defense-eliciting TMV-p50-U1 effector

To study the *in planta* dynamics of N and NbSPL6 association, we first determined the subcellular localization of these proteins. NbSPL6 contains a bipartite nuclear localization sequence (Figure S1). Transient expression of NbSPL6 fused to citrine under the control of a constitutive 35S promoter in *N. benthamiana* leaves confirmed that it localizes to the nucleus (Figure 1B and C). We further confirmed these results by biochemical fractionation. NbSPL6 fused to an HA tag was expressed in *N. benthamiana* leaves. NbSPL6-HA was detected exclusively in the nuclear-enriched (NE) fraction (Figure 1D). Similar biochemical fractionation experiments using previously characterized genomic N fused to a TAP tag (gN-TAP) [16], [17] indicated that N is present in both the cytoplasm (nuclear depleted, ND) and the nuclear (NE) fractions in the presence and absence of the p50-U1 viral effector (Figure 1E).

We next tested the association of NbSPL6 with N *in planta*. As a control, in these experiments, we used p50 from the TMV-Ob strain that does not elicit an N immune response. Previous attempts to localize p50-Ob described in [14] produced aberrant chloroplast localization [17]. However, extension of p50-Ob by six amino acids at the N-terminus produced nuclear and cytoplasmic localization, which is identical to that seen with tCFP-p50-U1 (Figure 1F). In agreement with previous reports [14], [23], p50-Ob-tCFP did not induce HR-PCD in N-containing *Nicotiana* plants while tCFP-p50-U1 induced HR (Figure 1G). The expression levels of the two p50 proteins were comparable (Figure 1H). For all further experiments, we used tCFP-p50-U1 as the elicitor of N-mediated immune response and p50-Ob-tCFP as the non-elicitor.

Low expression levels of NbSPL6-HA made it a challenge to detect the protein in total protein extracts. To overcome this problem, researchers working with Arabidopsis SPLs use miR156/157 resistant version of SPLs [20], [24]. We therefore created a miR resistant version of NbSPL6-HA (rNbSPL6-HA) that contains silent substitutions in seven nucleotides within the miRNA target site. rNbSPL6 is 100% identical to NbSPL6 at the amino acid level but resistant to miR156/157. When rNbSPL6-HA was transiently expressed in *N. benthamiana* leaves it accumulated to detectable levels in the total protein extracts (Figure 1I and Figure S2).

For *in planta* association experiments, we co-expressed gN-Myc and rNbSPL6-HA and 8 or 12 hours later tCFP-p50-U1 or p50-Ob-tCFP was infiltrated. The leaf samples were collected between 44 and 50 hours post-infiltration (hpi) of N and SPL6. As a control, gN-Myc was coinfiltrated with NLS-GUS-HA followed by infiltration with tCFP-p50-U1. Our results indicate that gN-Myc co-immunoprecipitates with rNbSPL6-HA only in the presence of defense eliciting tCFP-p50-U1 but not in the presence of the non-eliciting p50-Ob-tCFP (Figure 1I and Figure S2). gN-Myc failed to associate with NLS-GUS-HA even in the presence of tCFP-p50-U1 (Figure 1I and Figure S2). These results indicate that *in planta*, only p50-U1 activated N associates with NbSPL6.

### N and NbSPL6 associate within subnuclear bodies only in the presence of the defense-eliciting TMV-p50-U1 effector

**Figure 2:**
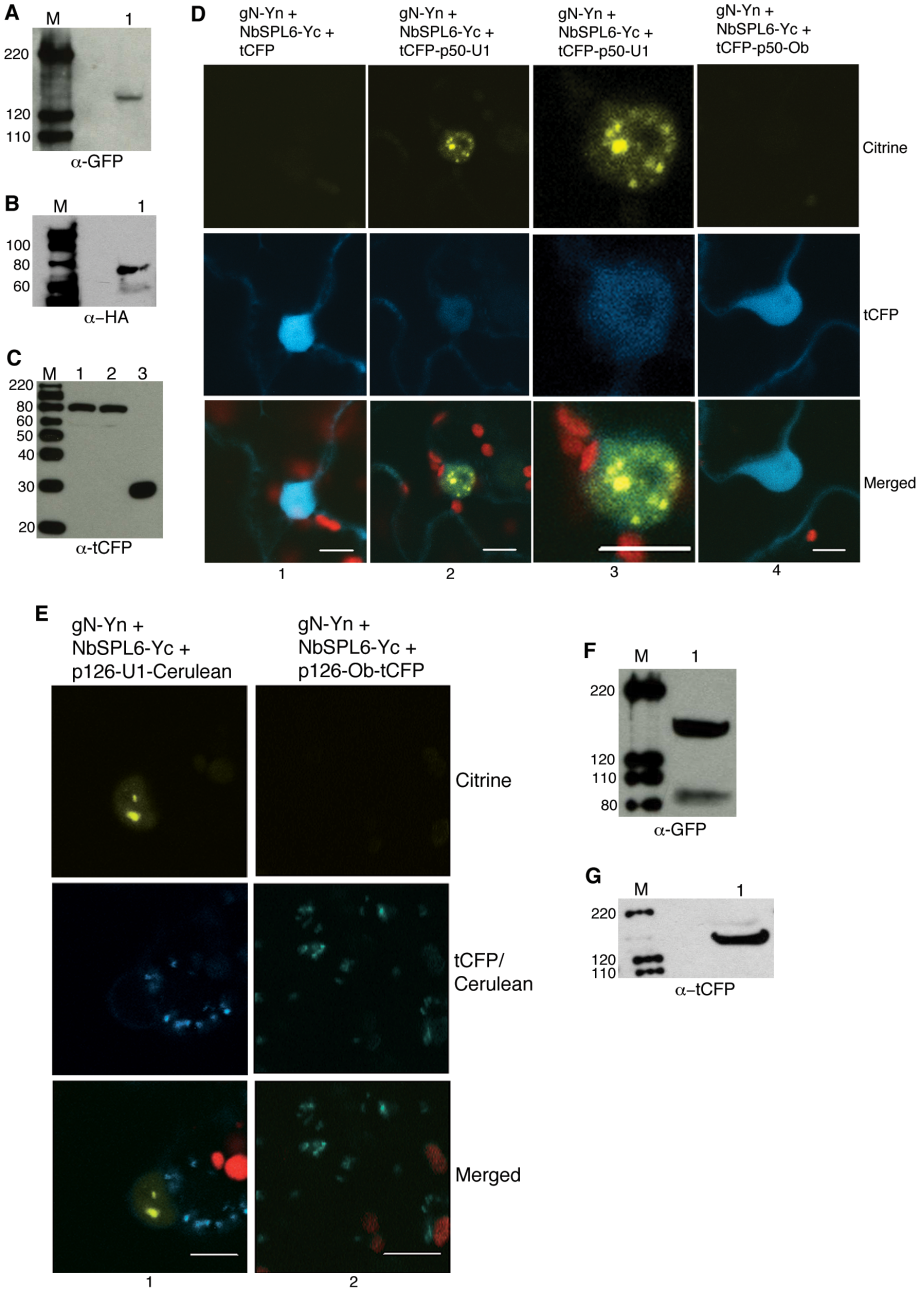
N associates with NbSPL6 in subnuclear bodies only during an active immune response. **A–C.** Western blots showing gN-Yn (A), NbSPL6-HA-Yc (B), tCFP-p50-U1 (C, lane 1), p50-Ob-tCFP (C, lane 2), and tCFP (C, lane 3). M indicates marker. Protein sizes marked on the left are in kD. **D.** Co-expression of gN-Yn and NbSPL6-Yc with tCFP did not reconstitute citrine fluorescence (column 1) in BiFC assays. However, co-expression of gN-Yn and NbSPL6-Yc with tCFP-p50-U1 resulted in the reconstitution of citrine fluorescence within subnuclear bodies (column 2 and 3). Images in the column 3 are magnified versions of the nucleus shown in column 2. Citrine fluorescence was not observed when gN-Yn and NbSPL6-Yc were co-expressed with the non-eliciting p50-Ob-tCFP (column 4). Scale bars = 10 µm. The red structures are chloroplasts. **E.** Co-expression of gN-Yn and NbSPL6-Yc in the presence of the full-length 126 kD TMV-U1 replicase (p126-U1-Cerulean) reconstituted citrine fluorescence (column 1). Citrine fluorescence was not observed in the presence of the non-eliciting 126 kD replicase from the TMV-Ob strain (p126-Ob-tagCFP) (column 2). Scale bar = 10 µm. The red structures are chloroplasts. **F and G.** Western blots showing p126-U1-Cerulean (F) and p126-Ob-tCFP (G). M indicates marker. Protein sizes marked on the left are in kD.

To further confirm N and NbSPL6 association during an immune response, we utilized the non-invasive Bimolecular Fluorescence Complementation (BiFC) assay [25]. We co-expressed genomic N fused to the N-terminal 155 amino acid residues of citrine (gN-Yn) and NbSPL6 fused to the C-terminal of citrine (NbSPL6-Yc). tCFP-p50-U1, p50-Ob-tCFP or tCFP was infiltrated 8–12 hrs after the initial infiltration. Expression of the full-length fusion proteins was confirmed by immunoblots (Figure 2A–C). Co-expression of gN-Yn and NbSPL6-Yc with the tCFP-p50-U1 effector reconstituted citrine fluorescence, indicating that following activation by p50-U1, N associates with NbSPL6 (Figure 2D, Columns 2 and 3). Interestingly, the reconstituted citrine fluorescence was localized to subnuclear bodies. In contrast, in the presence of the non-eliciting p50-Ob-tCFP, gN-Yn and NbSPL6-Yc failed to reconstitute citrine fluorescence in 87% of the cells examined (Figure 2D, column 4). Very weak citrine fluorescence was observed in the remaining 13% of the cells (based on the ratio of cells expressing fluorescence in the presence of p50-Ob-tCFP to that observed in the presence of tCFP-p50-U1). Similarly, co-expression of gN-Yn and NbSPL6-Yc with tCFP alone did not reconstitute citrine fluorescence in 90% of the cells examined (Figure 2D, Column 1). In 10% of the cells, we observed very weak citrine fluorescence. These results suggest that N predominantly associates with NbSPL6 within subnuclear bodies in the presence of the defense eliciting p50-U1 effector.

Since p50 is a part of the 126 kD TMV replicase, we tested for N and NbSPL6 association in the presence of the full-length 126 kD replicase. Consistent with previous data [26], p126-U1-cerulean localized to cytoplasmic bodies (Figure 2E, column1). Similar localization pattern was observed for p126-Ob-tCFP (Figure 2E, column 2). Expression of both the 126 kD proteins was confirmed by immunoblotting (Figure 2F and G). Co-expression of gN-Yn and NbSPL6-Yc in the presence of p126-U1-Cerulean reconstituted citrine fluorescence within subnuclear bodies (Figure 2E, column 1) but this was not observed in the presence of p126-Ob-tCFP (Figure 2E, column 2). These results confirm that N associates with NbSPL6 following its activation by the TMV-U1-replicase.

### NbSPL6 is required for N-mediated resistance to TMV

**Figure 3:**
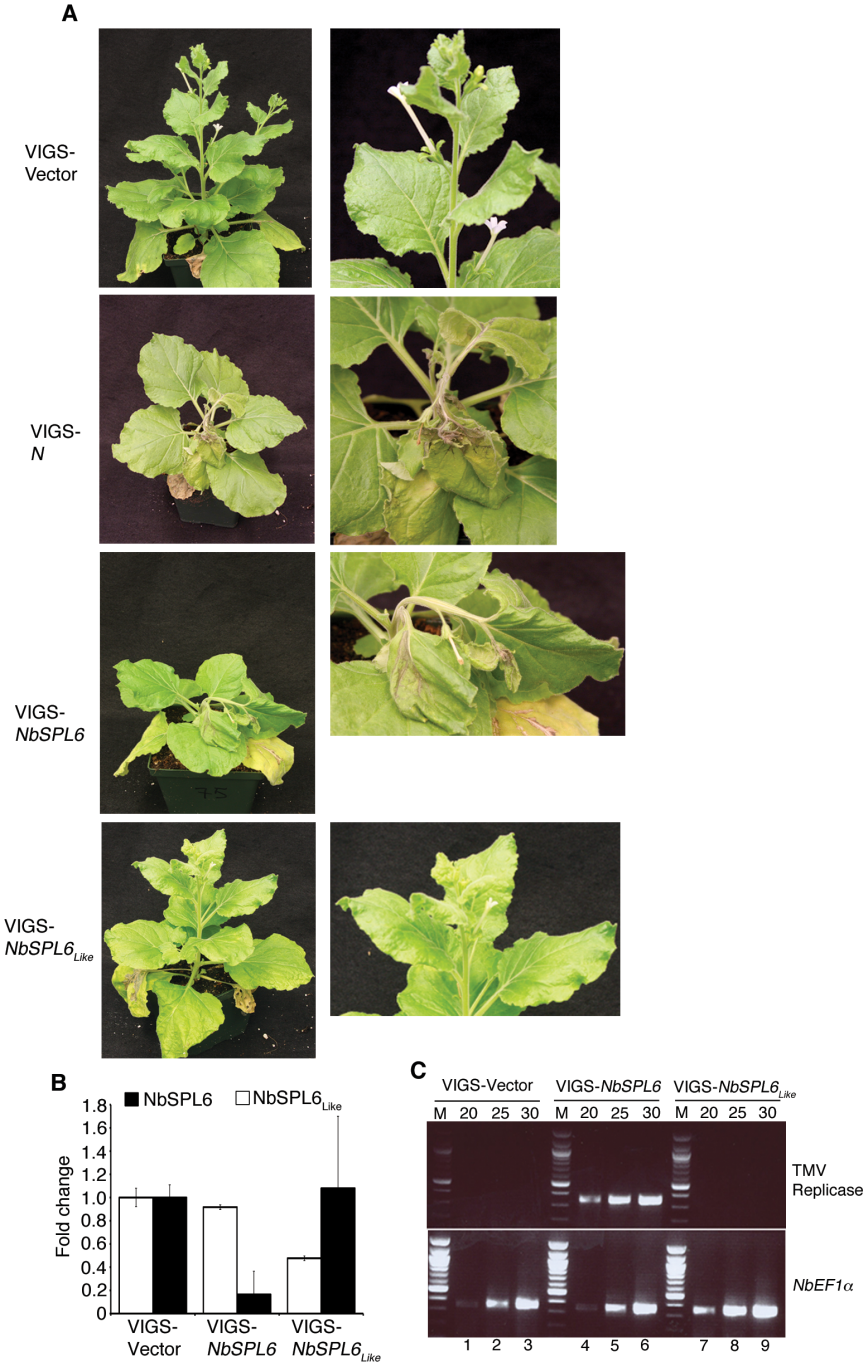
*NbSPL6* function is required for the N-mediated resistance to TMV. **A.** N-containing transgenic *N. benthamiana* plants were agro-infiltrated with an empty VIGS vector (VIGS-Vector), VIGS vectors designed to silence *N* (VIGS-*N*), *NbSPL6* (VIGS-*NbSPL6*) or *NbSPL6_Like_* (VIGS-*NbSPL6_Like_*). After 12 days, the plants were infected with TMV-U1 and monitored for the induction of the defense response. N-silenced plants and *NbSPL6*-silenced plants (second and third panels) were unable to restrict TMV-U1 and the virus spread to the systemic un-inoculated leaves. This is characterized by trailing necrosis and collapse of the shoot (second and third panels). The VIGS-Vector plants (top panels) and VIGS-*NbSPL6_Like_* (bottom panels) could evoke complete resistance against TMV-U1. The right panels are enlarged images of the systemic, un-inoculated leaves from each plant. **B.** qRT-PCR analysis showing relative *NbSPL6* (black bars) and *NbSPL6_Like_* (white bars) transcript levels in VIGS-Vector control, *NbSPL6* silenced plants and *NbSPL6_Like_*-silenced plants. Significant decrease in *NbSPL6* transcript levels but not *NbSPL6_Like_* transcript levels was observed in the *NbSPL6*-silenced plants. Similarly a significant decrease in *NbSPL6_Like_* transcript but not *NbSPL6* was observed in the VIGS- *NbSPL6_Like_* plants. Error bars = Std. Dev. **C.** The TMV 126 kD replicase transcripts were not detected in the upper un-inoculated tissue obtained from VIGS-Vector plants (rows 1–3) or VIGS-*NbSPL6_Like_* plants (rows 7–9) but were detected in VIGS-*NbSPL6* plants (rows 4–6). NbEF1α was used as the internal control. Numbers above the gel indicate PCR cycles. M = DNA marker.

We examined the function of NbSPL6 in the *N*-mediated resistance to TMV using a well-established *Tobacco rattle virus* (TRV)-based Virus Induced Gene Silencing (VIGS) approach [27]. This system has been successfully used to identify and characterize genes required for N-mediated resistance to TMV [16], [27], [28]. To test the function of NbSPL6 in *N*-mediated defense, we targeted the unique 3′ region of *NbSPL6* that includes the 3′UTR. Transgenic *N*-containing *N. benthamiana* plants [27] were inoculated with Agrobacterium-containing the recombinant TRV-*NbSPL6* and empty TRV-vector constructs. In addition, we also inoculated plants with the positive control, TRV-*N* that is designed to silence the *N* gene [27]. Twelve days post-silencing, the plants were infected with TMV-U1 and monitored for the induction of HR-PCD and resistance response. In the VIGS-vector control plants, TMV was restricted to the infection site and the upper uninoculated leaves remained healthy (Figure 3A, top panels; Figure S3). However, the *NbSPL6*-silenced plants exhibited a loss-of-resistance phenotype (Figure 3A, third panels; Figure S3). This is characterized by collapse of the inoculated leaf and movement of TMV into the systemic tissue eventually leading to spreading HR-PCD and death of the whole plant (Figure 3A, third panels; Figure S3). The *N* silenced plants showed a similar phenotype to the *NbSPL6*-silenced plants following inoculation with TMV (Figure 3A, second panels; Figure S3).

SPL family contains multiple members [21], [22]. The 70 amino acid SBP domain is conserved among different members while the region flanking the SBP domain is quite variable. To determine if loss of *N*-mediated defense to TMV is specific to NbSPL6, we silenced the *NbSPL6_Like_* gene. When compared to NbSPL6, NbSPL6*_Like_* shares 91% amino acid similarity within the SBP domain and 31% similarity at the full-length protein level (Figure S1). The phenotype observed for the *NbSPL6_Like_* silenced plants was similar to the vector control with the virus mainly being contained to the inoculated leaves (Figure 3A, bottom panels).

These experiments were repeated 3 times. We observed loss-of-resistance in 100% of the plants silenced for N and in 54% of plants silenced for *NbSPL6* (Figure S3). Quantitative real time RT-PCR (qRT-PCR) results showed that *NbSPL6* transcript levels reduced significantly in the VIGS-*NbSPL6* plants compared to the VIGS-vector control plants (Figure 3B). We did not observe a significant difference in the *NbSPL6_Like_* transcript levels between the VIGS-vector control and VIGS-*NbSPL6* silenced plants (Figure 3B). Similarly, in the VIGS-*NbSPL6_Like_* plants, *NbSPL6_Like_* transcript was downregulated but the levels of *NbSPL6* remained unchanged (Figure 3B). This indicates that the *NbSPL6* silencing effect is specific.

To confirm that TMV spreads systemically into the upper uninoculated leaves in the *NbSPL6*-silenced plants, we tested for the presence of the TMV transcripts in the upper un-inoculated leaves. A significant amount of TMV replicase RNA or coat protein RNA was detected in the *NbSPL6* and *N* silenced plants but not in the VIGS-vector control plants or VIGS-*NbSPL6_Like_* plants (Figure 3C; Figure S3). These results indicate that *NbSPL6* is required for *N*-mediated resistance to restrict TMV to the infection site.

### A functional P-loop within the NB domain of N is required for the association with NbSPL6

In a number of NB-LRRs including N, mutations within the P-loop of the NB domain have been shown to abolish functionality [29], [30]. It has been hypothesized that following effector recognition, the ATP binding/hydrolysis at the NB domain promotes a conformational change in the immune receptor, which shifts it into an active, signaling-competent state [30], [31]. Previously it was shown that a mutation in the lysine222 (gN^K222A^) or glycine221-lysine222 (gN^GK221-222AA^) residues led to a loss-of-function N protein [29], [32]. Since only activated N can associate with NbSPL6 (Figure 1 and 2), we tested the effect of P-loop mutations on this association.

**Figure 4:**
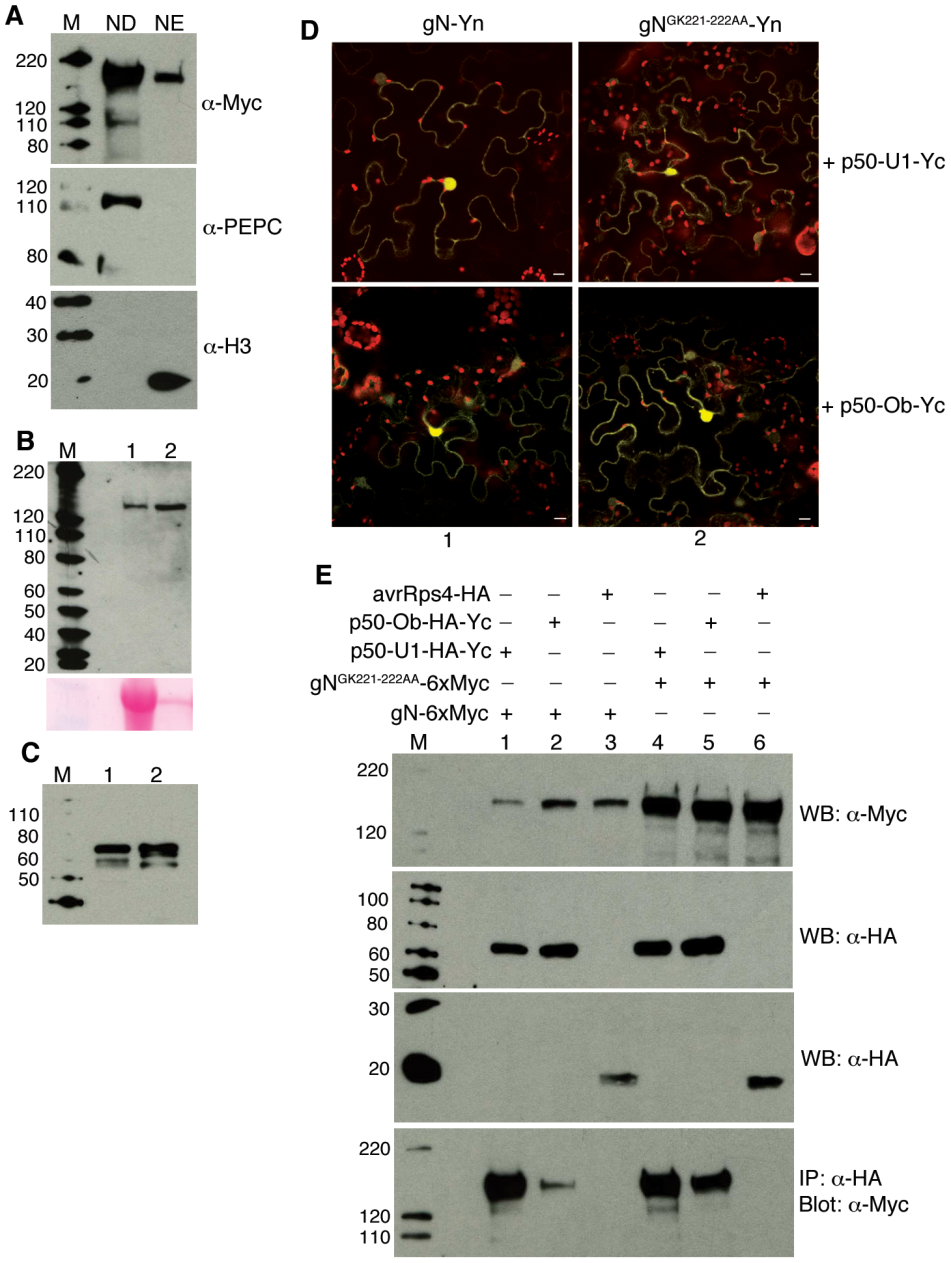
A P-loop mutant in the NB domain of the N immune receptor can still associate with p50-U1 or p50-Ob. **A.** Cellular fractionation of gN^GK221-222AA^-TAP expressing tissue shows that the mutant protein is present in both the cytoplasmic fraction (ND) as well as the nuclear enriched (NE) fraction (upper panel). PEPC was used as a cytoplasmic marker (middle panel) and Histone 3 (H3) was used as a nuclear marker (bottom panel). M indicates marker. Protein sizes marked on the left are in kD. **B–C.** Western blots showing the expression of gN-Yn (B, upper panel, lane 1), gN^GK221-222AA^-Yn (B, upper panel, lane 2), p50-U1-HA-Yc (C, lane 1) and p50-Ob-HA-Yc (C, lane 2). The input volume for N^GK221-222AA^-Yn (B, upper panel, lane 2) was adjusted to 1/20^th^ the volume loaded in lane 1 for gN (B, upper panel, lane 1). Ponceau staining (B, bottom panel) shows loading volume. M indicates marker. Protein sizes marked on the left are in kD. **D.** Co-expression of gN-Yn (column 1) or N^GK221-222AA^-Yn (column 2) with p50-U1- Yc (upper panels) and p50-Ob- Yc (lower panels) reconstitutes citrine fluorescence in BiFC assays. Scale bars = 10 µm. The red structures are chloroplasts. **E.** Co-immunoprecipitation of gN-6xMyc or gN^GK221-222AA^-6xMyc with p50-U1-HA-Yc, p50-Ob-HA-Yc or avrRps4-HA. Western blot analysis confirming expression of input proteins gN-6xMyc (panel1, lanes 1,2,3) and gN^GK221-222AA^-6xMyc (panel 1, lanes 4,5,6), p50-U1-HA-Yc (panel 2, lanes 1 and 4) and p50-Ob-HA-Yc (panel 2, lanes 2 and 5), and avrRps4-HA (panel 3, lanes 3 and 6). gN-6xMyc (panel 4, lanes 1 and 2) and gN^GK221-222AA^-6xMyc (lanes 4 and 5) co-immunoprecipitated with p50-U1 and p50-Ob. gN-6 myc or gN^GK221-222AA^-6xMyc did not co-immunoprecipitate with avrRps4 (panel 4, lanes 3 and 6). M indicates marker. Protein sizes marked on the left are in kD.

Biochemical fractionation experiments showed that gN^GK221-222AA^-TAP has a localization pattern similar to gN with the protein being observed in both the cytoplasm and nucleus (Figure 4A). BiFC assays were carried out to test for the association between the P-loop mutant gN^GK221-222AA^-Yn and p50-U1-Yc or p50-Ob-Yc. Expression of the proteins was confirmed by immunoblotting (Figure 4B and C). Interestingly, gN^GK221-222AA^-Yn accumulated to significantly higher levels compared to gN-Yn (Figure 4B), which is consistent with a previous observation [29], [32]. We observed reconstitution of citrine fluorescence when gN-Yn and gN^GK221-222AA^-Yn was co-expressed with p50-U1-Yc or p50-Ob-Yc (Figure 4D).

To further confirm the BiFC results, we performed co-immunoprecipitation assays. We transiently co-expressed gN-Myc or gN^GK221-222AA^-Myc with p50-U1-HA-Yc or p50-Ob-HA-Yc in *N. benthamiana* leaves. *Pseudomonas syringae* effector avrRps4-HA that is not recognized by N was used as a control. Both N and N^GK221-222AA^ associated with p50-U1 and p50-Ob though the association with p50-Ob was weaker (Figure 4E). N and N^GK221-222AA^ did not associate with avrRps4-HA (Figure 4E).

**Figure 5:**
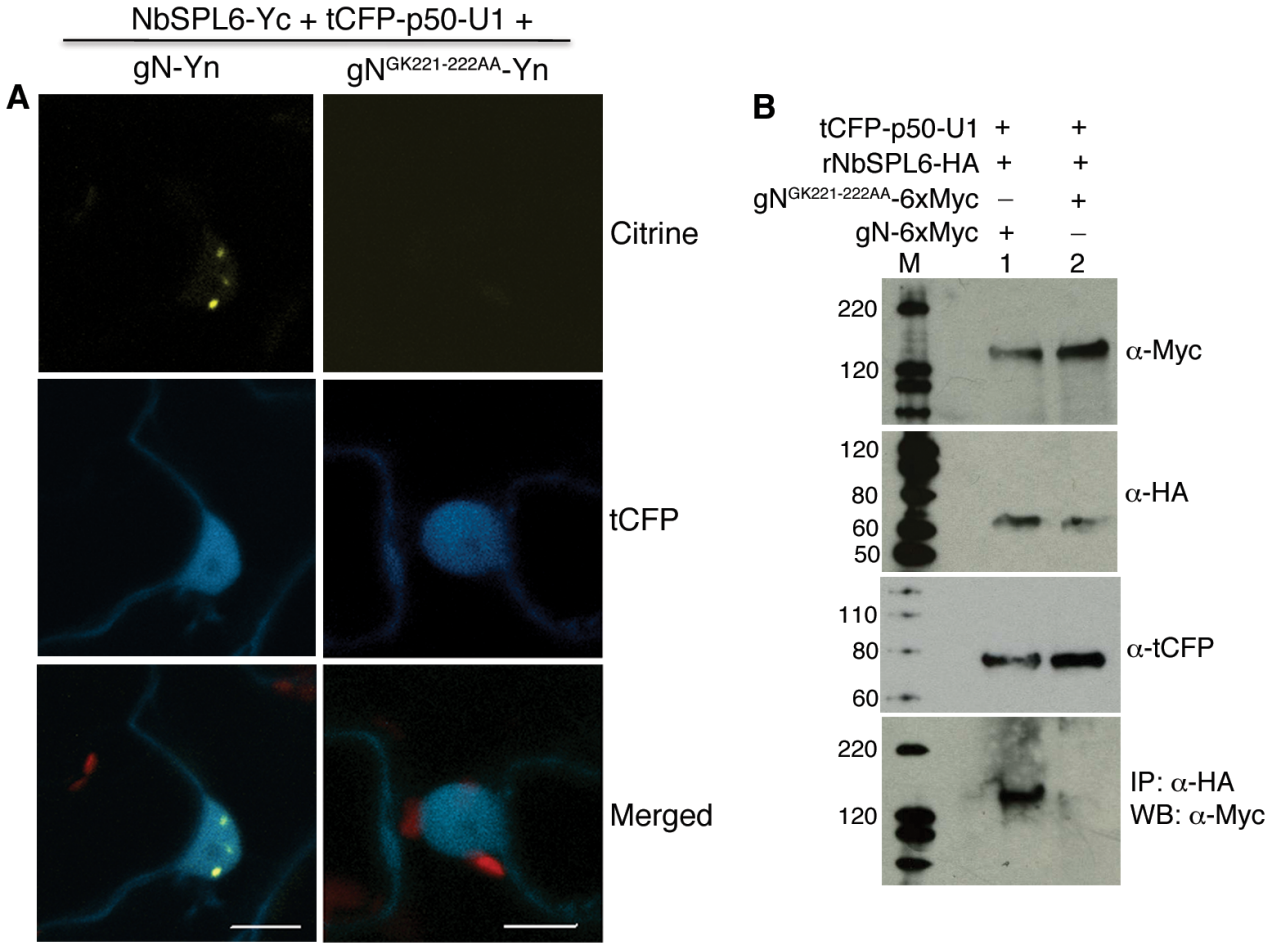
Mutation in the P-loop of the N immune receptor abolishes its association with NbSPL6. **A.** BiFC assay showing that gN-Yn when co-expressed with NbSPL6-Yc reconstitutes citrine fluorescence in the presence of the defense eliciting tCFP-p50-U1 effector (left columns). gN^GK221-222AA^-Yn when co-expressed with NbSPL6-Yc fails to reconstitute citrine fluorescence in the presence of tCFP-p50-U1 (right columns). Scale bar = 10 µm. The red structures are chloroplasts. **B.** gN^GK221-222AA^-6xMyc is unable to co-immunoprecipitate with rNbSPL6-HA in the presence of tCFP-p50-U1. Western blot analysis confirmed expression of input proteins gN-6xMyc and gN^GK221-222AA^-6xMyc (panel 1), rNbSPL6-HA (panel 2), and tCFP-p50-U1 (panel 3). While gN-6Myc co-immunoprecipitated with rNbSPL6 in the presence of p50-U1 (panel 4, lane 1), gN^GK221-222AA^-6xMyc failed to co-immunoprecipitate with rNbSPL6 (panel 4, lane 2). M indicates marker. Protein sizes marked on the left are in kD.

Since the N^GK221-222AA^ mutant fails to initiate the defense response in the presence of p50-U1, we analyzed the association of the mutant with NbSPL6. For this, gN^GK221-222AA^-Yn and NbSPL6-Yc were co-expressed in the presence of tCFP-p50-U1 using conditions similar to those used for gN. Under these conditions, we were unable to observe reconstituted citrine fluorescence (Figure 5A), indicating that gN^GK221-222AA^ does not associate with NbSPL6. These results were further confirmed by co-immunoprecipitation assays. gN^GK221-222AA^-Myc failed to associate with rNbSPL6-HA in the presence of tCFP-p50-U1 (Figure 5B). Collectively, these results indicate that a functional P-loop is not required for N's association with the defense-eliciting p50-U1 but is crucial for its association with NbSPL6. The P-loop activity may directly enable association with NbSPL6 and/or it activates N which temporally precedes NbSPL6 association.

### Arabidopsis SPL6 is required for the TIR-NB-LRR RPS4 immune receptor function

**Figure 6:**
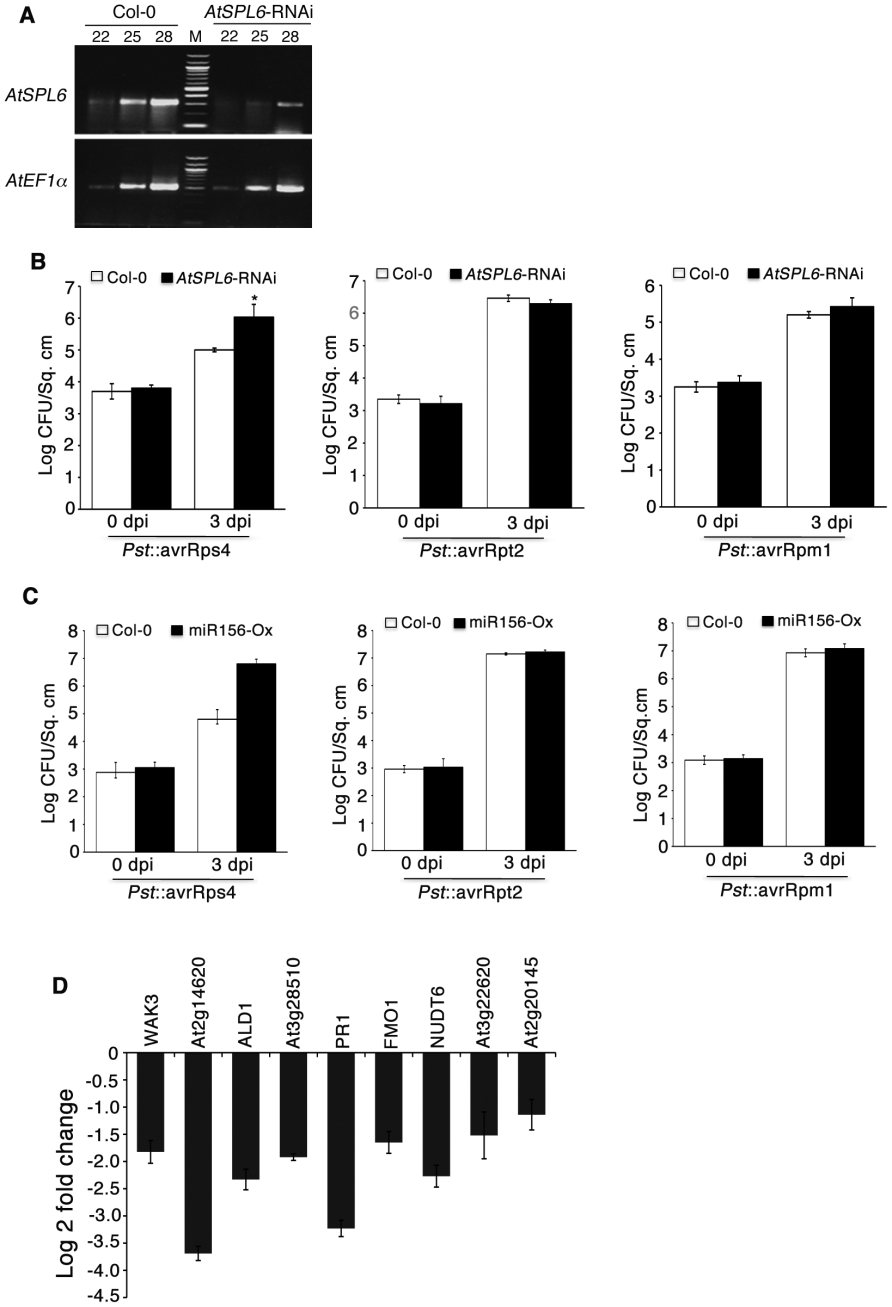
*AtSPL6* is required for RPS4-mediated defense against *Pst*::avrRps4. **A.** Semi-quantitative RT-PCR showing a reduction in *AtSPL6* transcripts in *AtSPL6*-RNAi plants (right) compared to Col-0 (left). *EF1α* was used as an internal control. Numbers above indicate PCR cycles. M = DNA marker. **B.** Bacterial growth on *AtSPL6*-RNAi plants. Col-0 and *AtSPL6*-RNAi plants were syringe infiltrated with 1×10^4^ CFU bacteria and titers determined at 0 and 3 dpi. RPS4-mediated resistance to *Pst*::AvrRps4 is compromised in *AtSPL6*-RNAi plants (left panel). RPS2- and RPM1-mediated resistance against *Pst*::AvrRpt2 (middle panel) and *Pst*::AvrRpm1 (right panel) is not compromised in the *AtSPL6*-RNAi plants. Error bars represent SD. *Two-tailed T test determined the difference to be statistically significant. Alpha = 0.05. **C.** Bacterial pathogen growth on miR156 overexpression plants. RPS4-mediated resistance to *Pst*::AvrRps4 is compromised in plants overexpressing miR156 (miR156-OX) (left panel) but not RPS2- and RPM1-mediated resistance against *Pst*::AvrRpt2 (middle panel) and *Pst*::AvrRpm1 (right panel). Error bars represent SD. **D.** Quantitative Real time PCR analysis of transcript levels of selected genes (indicated on top of the graph) whose expression is significantly lower in *AtSPL6*-RNAi lines compared to Col-0 during RPS4-mediated immune response in whole genome microarray analysis (see results section for details). The log2 fold change reduction in corresponding gene expression in *AtSPL6*-RNAi plants compared to Col-0 is plotted. Error bars represent SD.

Characterization of SPLs in Arabidopsis, rice and *Antirrhinum* revealed that SPLs have conserved function in development among different species (reviewed in [21], [22]). We therefore tested the role of Arabidopsis *SPL6* (the presumed ortholog of *NbSPL6*) in innate immunity. For this, first we analyzed SAIL_18b_C07 line (http://signal.salk.edu/cgi-bin/tdnaexpress) in which the T-DNA insertion is in the 3′UTR of *AtSPL6*. RT-PCR analysis revealed that *AtSPL6* transcript levels are similar in the insertion line and the wild type Col-0 plants (data not shown). We therefore generated *AtSPL6* RNAi lines. After characterization of RNAi lines, we selected two independent lines (#3 and #9) that showed significant reduction in *AtSPL6* RNA levels (Figure 6A; Figure S4A).

In Arabidopsis Col-0 plants, the TIR-NB-LRR RPS4-mediates defense against *Pseudomonas syringae* pv tomato (*Pst*) expressing the avrRps4 effector (*Pst*::avrRps4). In agreement with previously published report [33], an *rps4* knockout line (*rps4-2*) shows significant susceptibility to *Pst*::avrRps4, (Figure S4B). We observed a 10 fold increase in *Pst*::avrRps4 titer in two independent *AtSPL6*-RNAi lines compared to Col-0 infected plants (Figure 6B, Figure S4B).

We also tested, if AtSPL6 function is required for CC-NB-LRRs RPM1 and RPS2 in Col-0 that provide resistance against *Pst*::avrRpm1 and *Pst*::avrRpt2 respectively. In contrast to *Pst*::avrRps4, there was no difference in the growth of *Pst*::avrRpm1 and *Pst*::avrRpt2 between *AtSPL6*-RNAi and Col-0 plants (Figure 6B). Similarly growth of the virulent pathogen *Pst* DC3000, that evokes only the basal immune response, was found to be similar in the *AtSPL6*-RNAi lines and Col-0 (Figure S4C). These results indicate that *AtSPL6* is required for the TIR-NB-LRR RPS4-mediated immunity but not for CC-NB-LRR RPM1, RPS2 function or basal immunity.

In Arabidopsis, 11 *SPL* genes including *AtSPL6* are regulated by miR156 [20]. In miR156 overexpression (miR156-OX) plants, whole genome microarray experiments revealed that the transcript levels of all targeted *SPLs* including those of *AtSPL6* are down-regulated [19]. RPS4 expression level remained unaltered in these plants. Interestingly, *Pst*::avrRps4 grew to ∼20 fold higher titer in miR156-OX plants compared to Col-0 (Figure 6C). However, there was no effect on the RPS2- and RPM1-mediated defense response (Figure 6C). These pathogenicity assays confirm that AtSPL6 is required for RPS4-mediated defense response against *Pst*::avrRps4.

### AtSPL6 may regulate RPS4-mediated defense responsive genes

Since our results indicated that SPL6 is required for the function of two nuclear-localized TIR-NB-LRRs from two different plant species, we reasoned that it might participate in transcriptional reprogramming during an immune response. Whole transcriptome microarray analysis is well established in Arabidopsis, so we performed microarray analysis of *AtSPL6*-RNAi plants using Affymetrix ATH1 Arabidopsis GeneChips. Col-0 and *AtSPL6*-RNAi plants were either mock-inoculated with 10 mM MgCl_2_ or inoculated with *Pst*::avrRps4 (10^7^ cfu/ml) and tissue was collected at 3 h and 6 h post-infection. These time points and conditions were chosen based on similar whole genome microarray analysis carried out on *Pst*::avrRps4 infected Arabidopsis [34],[35]. When compared to Col-0, our analyses identified 312 and 387 genes that were expressed at a lower level (2 fold or more) at 3 hpi and 6 hpi respectively in the *AtSPL6*-RNAi plants. Moreover, of the 2678 genes that were activated during RPS4-mediated response in Col-0, a total of 322 genes remained unresponsive in the *AtSPL6*-RNAi plants (Table S1).

Biological Networks Gene Ontology (BINGO) [36] analysis of AtSPL6 regulated genes revealed a strong enrichment of defense genes (GO defense response genes, Cor P value = 5.14E-11). Some of these genes include previously characterized defense responsive genes such as *PR1*, *ALD1*, *AIG1*, *NUDT6*, *PAD4*, *FMO1*, and *LURP1* [34], [37]–[39] (See Table S1). We picked a small subset of candidate genes from our microarray data set and carried out quantitative real-time PCR to confirm their responsiveness to *Pst*::avrRps4 infection in Col-0 and *AtSPL6*-RNAi plants. This set included genes that have previously been shown to be responsive during RPS4-mediated resistance [34]. qRT-PCR confirmed that in *AtSPL6*-RNAi plants, the 9 selected genes were less responsive to *Pst*::avrRps4 (Figure 6D). Together, these results indicate that SPL6 transcription factor functions as a positive regulator of defense gene induction during innate immunity. Future experiments will be directed towards identifying the direct targets of SPL6 during innate immunity.

## Discussion

**Figure 7:**
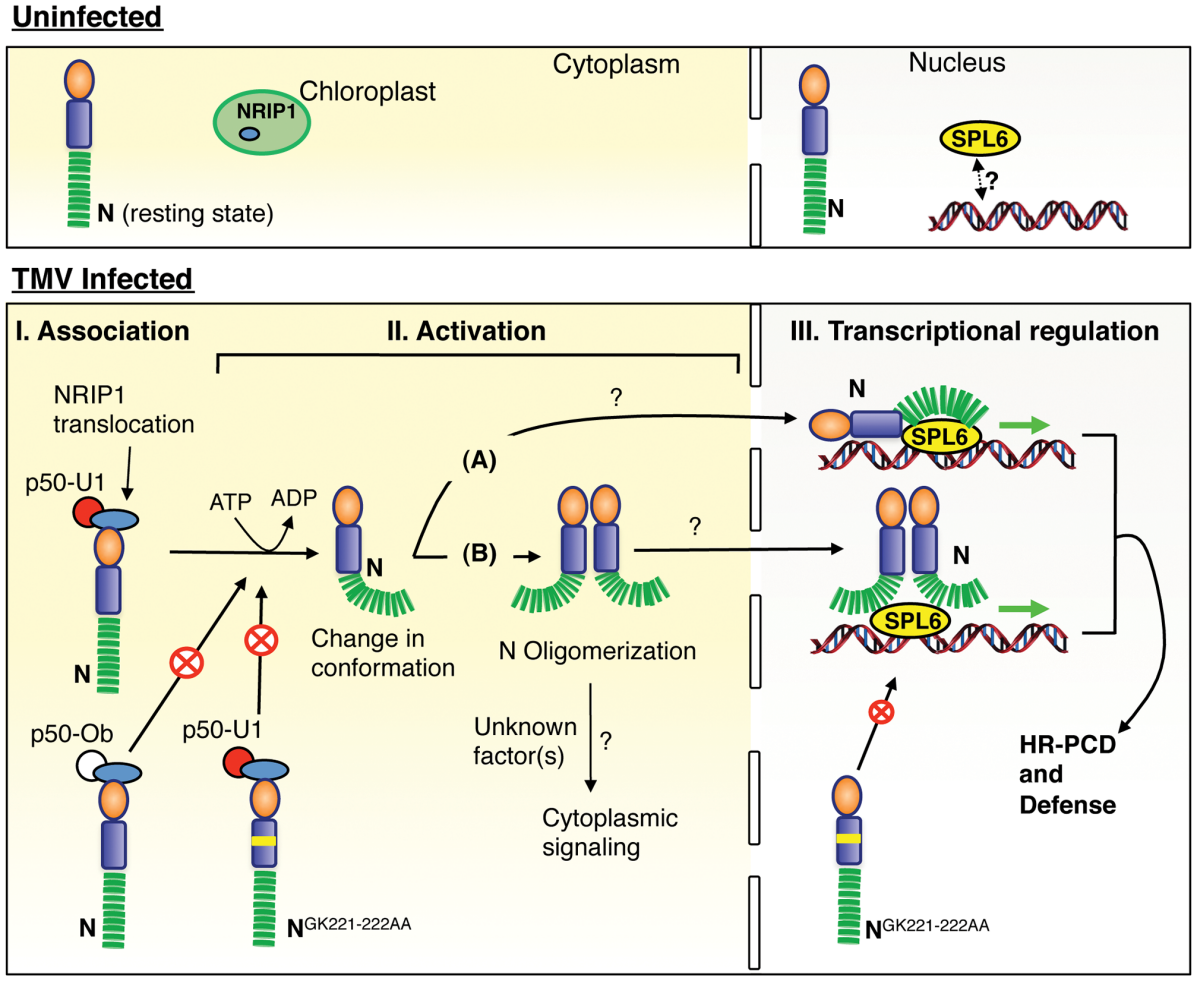
Model for immune receptor-mediated recognition of pathogen and resultant defense gene activation. In uninfected cells, nuclear N does not associate with SPL6; as a result defense genes are not transcribed. Following TMV infection, there are 3 distinct phases for successful activation of a defense response. In phase I (Effector association), the viral effector promotes the relocalization of chloroplast NRIP1 into the cytoplasm and the p50-U1 and NRIP1 complex associates with N. This ternary complex could, by an as yet unknown mechanism, promote an ATP-dependent conformational change in N potentiating it for further signaling events. The N^GK221-222AA^ P-loop mutant can associate with p50-U1 but is unable to undergo the conformational change and hence is not activated. p50-Ob from the non-eliciting TMV-Ob strain can also associate with N, but may not be able to induce a conformational change. Phase II (Activation) - The ATP bound N may associate with nuclear SPL6 (pathway A) thereby activating defense gene expression. Alternately, N undergoes TIR domain-mediated oligomerization leading to recruitment of unknown host protein(s) that activate(s) N. This oligomerized N complex may associate with nuclear SPL6 (pathway B). In phase III (transcriptional regulation), activated N associates with SPL6. This either enhances SPL6 interaction with the specific defense responsive gene promoters or leads to recruitment of transcription machinery. The end result is the transcription of key immune response genes whose products are required for efficient induction of HR-PCD and defense.

We have identified, for the first time, a novel conserved role for the SPL6 transcription factor in innate immunity. We provide evidence to show that it is a key nuclear partner that aids defense responses mediated by N and possibly RPS4 TIR-NB-LRR immune receptors. We show that SPL6 is required for N-mediated resistance to TMV in *Nicotiana*. N and SPL6 associate *in planta*, within subnuclear bodies only during an active immune response. SPL6 is also required in Arabidopsis for the induction of defense by the nuclear-localized TIR-NB-LRR RPS4 but not for defense mediated by plasma membrane localized RPM1 and RPS2. Preliminary gene regulation assay suggest that SPL6 is a positive regulator of defense. Thus, SPL6 plays a conserved role in the TIR-NB-LRR mediated immune response across different plant species. Based on our data, we present a model for N-mediated immune response activation that details pathogen recognition by N in the cytoplasm followed by its activation and subsequent regulation of defense genes through nuclear SPL6 activity (Figure 7).

### SPL6 is the first member of SPL family known to play a role in innate immunity

SBP-box containing genes are ubiquitously found in the plant kingdom and a number of Arabidopsis SPLs (SPL3, SPL4, SPL5, SPL9 and SPL15) have been found to have overlapping functions especially in regulating flowering time, leaf development, and transition from juvenile to adult phase [21]. While the role of SPLs in development has been extensively studied, their role in defense has not been described. Our report on SPL6 is the first to show a transcriptional regulatory role for the SPL family in innate immunity. Future studies should determine how SPL6 participates in defense transcriptome induction. In addition, possible role(s) for other SPLs in plant innate immunity should be investigated.

### Immune receptor association with the effector is not sufficient to activate immune response

N provides resistance against all strains of TMV except TMV-Ob, hence at temperatures above 20°C, TMV-Ob can systemically infect N-containing plants [13]. Initial attempts to characterize p50-Ob were complicated by the fact that the protein mislocalized to the chloroplast [17]. We therefore used a p50-U1-Ob chimera, which had a localization pattern similar to p50-U1 (cytoplasm and nuclear localization) [16], [17]. While we could not detect an association between the p50 chimera and N, we observed that it could still associate with, and alter the localization of NRIP1 [16]. Here, we have used p50-Ob with six additional amino acids at the N-terminus. The localization pattern of this p50-Ob is similar to that of N eliciting p50-U1. N can associate with p50-Ob though the association is weaker than that seen with p50-U1. However, this association is not sufficient to trigger N-mediated HR-PCD and defense. Our results indicate that this could partly be because of Ns failure to associate with SPL6 in the presence of p50-Ob. Therefore, immune receptor association with the pathogen effector alone is not sufficient to induce an immune response. We hypothesize that in the case of N and p50-U1, following association, the N-NRIP1-p50-U1 complex promotes a crucial conformational change in N that enables it to perform the subsequent steps necessary for defense signaling. N may be unable to undergo such a conformational change in the presence of p50-Ob, making the association unproductive. We envision that N activation is dependent on the structural features of p50-U1 that are different in p50-Ob. This is in agreement with previous studies using p50-U1-Ob chimeras and mutational analysis that have indicated that the three dimensional structure of TMV p50 is more important in HR-PCD induction than the primary sequence [14]. [23] showed that a single P1089L point mutation in the p50 domain of TMV-Ob (p50-Ob-NL-1) was sufficient to restore N recognition, and proposed that this mutation might alter the structural conformation of the p50 domain to enable N activation. In agreement with this, a preliminary structural analysis predicted that the leucine at position 1089 results in a protein containing a single long α helix in the place of two α helices [23]. Detailed structural analysis of p50-U1, p50-Ob and p50-ObNL-1 is necessary to gain insights into the importance of effector structure and its role in N activation and defense signaling.

NRIP1 localizes to the nucleus following association with p50 but it is unclear if it associates with SPL6 or is a part of a complex with N and SPL6. We also observed a consistent enhancement in N protein accumulation in tissue specifically co-expressing N and p50-U1. This is in agreement with previous observations [29], [32]. Interestingly the levels of N protein appear to increase mainly in the nuclear-depleted tissue. It is possible that cytoplasmic N protein may be stabilized during an active immune response and further experiments are needed to address this hypothesis.

### Functional nucleotide binding of N is crucial for its association with the SPL6 transcription factor

The P-loop within the NB domain of plant NB-LRRs is the site of ATP binding [30]. Mutations in the P-loop of N are predicted to abolish its ATP binding ability. In agreement with this, P-loop mutants of N lose resistance to TMV [29], [32]. Our biochemical fractionation experiments indicate that ATP binding is not the major factor that determines nuclear localization since gN^GK221-222AA^ has a localization that is similar to gN. These results are similar to the observations made with RPS4 [33] but different from CC-NB-LRR Rx in which a P-loop mutation significantly reduced its nuclear accumulation [40]. ATP binding is also not necessary for N association with the p50 effector since N^GK221-222AA^ could associate with p50. Similarly the P-loop mutant of Arabidopsis TIR-NB-LRR RPP1 can associate with its cognate effector ATR1 from *Hyaloperenospora arabidopsidis* [41]. However, our results show that a functional P-loop is necessary for Ns association with SPL6 in the nucleus. It is possible that N may undergo an ATP binding/hydrolysis-dependent conformational change that switches inactive N into an activated, signaling competent state. It is only this activated N that can associate with SPL6 to induce a successful immune response.

### Only defense eliciting p50-U1 effector-activated N associates with SPL6

The results presented here point to N activation prior to its association with SPL6 in the nucleus. What events lead to N activation? It has previously been reported that N undergoes TIR domain-mediated oligomerization only in the presence of defense eliciting p50-U1 effector and that this process requires a functional P-loop [32]. It is possible that oligomerization is the crucial step that leads to N activation and that this must occur prior to SPL6 association. While the P-loop mutant N^GK221-222AA^ cannot oligomerize in the presence of p50-U1 [32], it is as yet unknown if p50-Ob can induce oligomerization of wild-type N. Future studies should test if the p50-Ob structural constraints discussed above limit N's ability to undergo oligomerization.

It is interesting that N associates with SPL6 within distinct subnuclear bodies. Certain plant MADS box transcription factors also associate in distinct subnuclear bodies [42]. The authors hypothesize that the subnuclear regions represent sites in the chromatin to which transcription factors are recruited. Localization of certain mammalian and nematode transcriptional co-regulators to nuclear bodies has also been documented [43], [44]. Thus it is possible that subnuclear bodies where N and SPL6 are associating may correspond to regions of active defense gene transcription.

### SPL6 is a positive regulator of defense gene expression

Silencing Nb*SPL6* in *Nicotiana* plants compromises N-mediated defense against TMV. Similarly, At*SPL6*-RNAi plants are compromised in RPS4-mediated resistance to Pst::avrRps4. These results suggest that SPL6 positively regulates immune signaling mediated by two different TIR-NB-LRRs from two different plant species. Our microarray analysis revealed that a significant number of RPS4-mediated defense responsive genes might be regulated, either directly or indirectly, by SPL6.

Our data suggest that N and possibly RPS4 function as positive regulators of defense genes by recruiting transcription factors like SPL6. This is similar to the mechanism used by the mammalian NLRs CIITA and NLRC5, which recruit transcription factors to induce the expression of MHCII and MHCI genes [10], [11]. The recruitment and modulation of SPL6 by N, WRKYs by MLA10 and TPR1 by SNC1 highlights not only the diversity of transcription factors that are regulated by immune receptors but also shows the different strategies used by immune receptors to activate defense gene expression.

The role, if any, of nuclear-localized immune modulator Enhanced Disease Susceptibility (EDS1) in N-SPL6 association needs to be investigated. EDS1 is required for basal immunity and for the function of TIR-NB-LRRs reviewed in [45]. EDS1 resides in cytoplasmic and nuclear pools and nuclear EDS1 is required for immune receptor-mediated induction of transcriptional reprogramming [35]. Activation of RPS4 in the presence of bacterial avrRps4 has been shown to enhance accumulation of EDS1 in the nucleus [35]. Recent evidence indicates that EDS1 associates with three TIR-NB-LRRs - RPS4, SNC1, and RPS6 in the cytoplasm and nucleus [46], [47]. Future research will be directed towards testing for possible requirement of EDS1 in modulation of SPL6 activity.

### Model for N-mediated recognition of TMV and resultant activation of gene expression

Given these data, we propose the following model that details the molecular events from pathogen recognition to transcriptional reprogramming (Figure 7). In uninfected cells, N is in its resting state and found in the nucleus and in the cytoplasm. For several immune receptors such as Rx, Bs2, Mi, I2, and RPS5, extensive intra-molecular interactions keep the protein in an auto-inhibited state (reviewed in [31]). However, similar interactions have not been shown to occur with N *in planta* [32]; Dinesh-Kumar, unpublished). Alternatively, unknown host factor(s) may associate with N to keep it in an inhibitory state. In uninfected tissue, NRIP1 is solely localized to the chloroplast [16], and nuclear N and SPL6 do not associate. SPL6 may associate with the cis-acting elements of defense responsive genes, however, they are not transcriptionally active.

During TMV infection, the presence of the viral p126 replicase or the p50 effector induces NRIP1 relocalization from the chloroplast to the cytoplasm and nucleus (not shown in the model). In the cytoplasm, NRIP1 associates with p50/p126 and this complex is recognized by cytoplasmic N (Figure 7, phase I). The initial events in effector association do not seem to depend on functional P-loop because the N^GK221-222AA^ mutant can still associate with p50 (Figure 7, phase I). However, following effector association, we hypothesize that p50-U1 alters the structure of N to induce a conformational change that would require ATP binding and/or hydrolysis. Alternatively, there may be a secondary interaction between the LRR domain and p50-U1 that may release the TIR-NB interface to facilitate nucleotide binding [31], [48]. Even though N is not fully activated, this step ‘potentiates’ N for further interaction/signaling events (Figure 7, phase II). The P-loop mutation, which abolishes ATP binding, would preclude the conformational change and the protein would remain inactive (Figure 7 phase I). Although p50-Ob can associate with N, it may be that p50-Ob does not induce the crucial conformational change, ATP binding/hydrolysis, and/or oligomerization necessary for subsequent defense-signaling steps (Figure 7, phase I). As a result, p50-Ob bound N is unable to switch into an activated state or associate with SPL6 in the nucleus (Figure 7, phase I).

It is as yet unclear as to whether the conformational change induced in N is sufficient for it to bind to nuclear SPL6. If this were the case, then potentiated N would directly translocate into the nucleus to bind with SPL6 and enhance the transcriptional activation of defense responsive genes (Figure 7, phase II-pathway A). Alternatively, additional steps may be required before N can associate with SPL6. For example, following the potentiation step, the TIR domain of N may mediate oligomerization. ATP binding is crucial to this step since the P-loop mutant is unable to undergo oligomerization [32]. However, oligomerzation is not sufficient to make N signaling-competent since some TIR and NB domain mutants that can oligomerize still fail to elicit HR-PCD [32]. Thus the oligomerization step may lead to the recruitment of additional host factor(s) that then assist N into attaining its final signaling competent state (Figure 7, phase II). The oligomerized and activated N translocates into the nucleus to associate with SPL6 (Figure 7, phase II pathway B). To distinguish between these two pathways, it must be determined which form of N (activated monomeric N or oligomerized N) is capable of binding SPL6.

Within the nucleus, activated N associates with SPL6 to either enhance its DNA binding abilities or to recruit the transcriptional machinery to the SPL6 bound promoters. In either event, N and SPL6 association is the key step towards transcription of defense genes (Figure 7, phase III).

In conclusion, results presented here lend support to the emerging concept that nuclear-localized plant immune receptors directly regulate defense genes by controlling the activity of key transcription factors. It highlights the remarkable ability of immune receptors to recognize pathogens as well as to regulate nuclear activities.

## Materials and Methods

### Nuclear fractionation

Nuclear fractionation was performed using a modified protocol described by [49]. Plant tissue was gently ground in modified Honda buffer (2.5% Ficoll 400, 5% Dextran T40, 0.4M Sucrose, 25 mM Tris-HCl, pH 7.4, 10 mM MgCl_2_) and complete protease inhibitor cocktail (Roche) in a mortar and pestle. The ground tissue was filtered through 70-µm nylon mesh. Triton X-100 was added to a final concentration of 0.5% and the tissue was incubated on ice for 15 minutes. The lysate was centrifuged at 100 g for 5 minutes to remove cellular debris followed by centrifugation at 1500 g to precipitate the nuclei. An aliquot of the supernatant was collected for the Nuclei Depleted fraction. The nuclei enriched pellet was washed 3 times in Honda buffer containing 0.1% Triton X-100. The pellet was resuspended in an appropriate volume of Nuclei sonication buffer (1 mM EDTA pH 8.0, 10%v/v glycerol, 75 mM NaCl, 0.05% w/v SDS, 100 mM Tris HCl, pH 7.4, 0.1% Triton X-100) with complete protease inhibitor (Roche)) and sonicated 4 times (10 s at 20% capacity). The sonicated samples were centrifuged at 12,000 g for 30 min at 4°C and the supernatant was collected as the Nuclei Enriched fraction.

### Protein expression analysis

*Agrobacterium tumefacians* strain GV2260 containing different expression constructs were infiltrated into *N. benthamiana* leaves as described previously [16]. N and N^GK221-222AA^ containing cultures were adjusted to OD_600_ = 2.1; NbSPL6 to OD_600_ = 1.5; TMV-126 kD to OD_600_ = 1.2; and p50 to OD_600_ = 1. For co-infiltration assays, N and NbSPL6 cultures were mixed in a 1∶1 proportion and infiltrated into 4-week old *N. benthamiana* leaves. 8 to 12 hrs post infiltration, p50 or TMV-126 kD cultures were infiltrated into the same leaf sectors.

Plant tissue expressing the protein(s) of interest was collected and ground in liquid nitrogen. Total protein extracts were prepared and immunoblots were probed and processed as previously described [16]. Antibodies used include mouse anti-cMyc (Santa Cruz) or mouse anti-cMyc-peroxidase (Roche), mouse anti-GFP (Covance), rabbit anti-tCFP (Evrogen), rat anti-HA (Roche) or rat anti-HA peroxidase (Roche), rabbit anti-PEPC (Rockland), rabbit anti-Histone H3 (Abcam) and anti-mouse, anti-rat or anti-rabbit peroxidase (Sigma). In the blots that were probed with anti-Myc or anti-HA peroxidase, the PVDF membrane section containing the protein markers was probed separately with anti-rabbit peroxidase.

### Co-immunoprecipitation assays

For co-immunoprecipitation assays with N and NbSPL6, Agrobacterium containing the different expression constructs were infiltrated into *N. benthamiana* leaves as described previously [16], [17]. N and N^GK221-222AA^containing cultures were adjusted to OD_600_ = 2.1; SPL6 to OD_600_ = 1.5; NLS-GUS-HA and p50 (U1 and Ob) to OD 1.0. Plant tissue was ground in liquid nitrogen and the proteins were extracted using the co-immunoprecipitation buffer (100 mM NaCl, 20 mM Tris pH 7.5, 1 mM EDTA, pH 8.0, 0.1% Triton, 10% Glycerol, 5 mM DTT, 2 mM NaF, 1 mM PMSF) and Complete protease inhibitor cocktail (Roche). The extracts were centrifuged at 3000 g for 5 minutes and the supernatant was passed through a Qiashredder column (QIAGEN) to remove residual cell debris. The filtrate was pre-cleared with protein G sepharose beads (Amersham Bioscience) with a 30 min incubation at 4°C. The samples were centrifuged at 3000 g for 2 minutes and anti-HA agarose beads (Sigma-Aldrich) were added to the supernatant. The samples were rotated for 2 hrs at 4°C and washed 3 times with co-immunoprecipitation buffer containing 200 mM NaCl. The beads were boiled with 2× loading buffer and samples were separated on an SDS-PAGE gel followed by western blotting.

For Immunoprecipitation assays with N and p50, Agrobacteria containing the different expression constructs were infiltrated into *N. benthamiana* leaves as described above. avrRps4 containing cultures were infiltrated at an OD_600_ = 1.0. The ground plant tissue was extracted with co-immunoprecipitation buffer containing 150 mM NaCl. The samples were centrifuged at 20,817 g for 10 minutes. The supernatant was centrifuged at 20,817 g for 5 min to remove residual cell debris. The samples were processed as mentioned above, the only difference being that the wash buffer contained 300 mM NaCl and 0.2% Triton. The samples were washed 4 times.

### Fluorescence microscopy

*Agrobacterium* containing the different constructs were infiltrated into *N. benthamiana* leaves at the ODs indicated above. Live plant tissue imaging was performed using a Zeiss LSM510 META confocal microscope (Carl Zeiss) using 40× or 63× apochromatic water immersion objectives. For tissues expressing N, SPL6, p50 and p126, samples were visualized for protein expression between 44 to 50 hrs post N and SPL6 infiltration. All other tissue samples were visualized 44 hrs post infiltration. The 458 nm and 514 nm excitation laser lines of a 25 mW Argon laser (Coherent) with appropriate bandpass emission filters were used to image citrine, tCFP, and cerulean. The 458 nm laser line of a 25 mW Argon laser and a META detector were used for imaging chloroplast autofluorescence. For BiFC assays, percentage of cells expressing citrine fluorescence was determined in 5 mm sq tissue sectors.

### VIGS assay

VIGS assays were carried out on transgenic N-containing *N. benthamiana* plants as described previously [27]. 12 days post silencing, two leaves from each plant were mechanically inoculated with diluted TMV-U1-infected leaf extract. The plants were monitored for the development of HR-PCD and systemic infection up to 14 days post TMV infection. VIGS assay was repeated three times using up to a total of 30 plants per VIGS construct.

### Characterization of *AtSPL6* T-DNA insertion line - SAIL_18b_C07

SAIL_18b_C07 seeds were obtained from ABRC and confirmed for the presence of the T-DNA insertion using the LB primer AGA TGA AGA CGA CCA CCG TAC and RB primer TGT TGC AGA AAA TGA TGT TGC along with LB1 T-DNA primer GCC TTT TCA GAA ATG GAT AAA TAG CCT TGC TTC C. Total RNA from homozygous insertion plants and Col-0 was isolated using RNeasy Mini kit (QIAGEN). 3 µg of RNA was used for the synthesis of cDNA using SuperScript II reverse transcriptase (Invitrogen). Semi quantitative PCR was performed as described previously [27] using *AtSPL6* and *EF1α* specific primers.

### Generation and characterization of *AtSPL6*-RNAi lines

The primer pair 5′CGG CTG GGT ACC GTT TCA TTT CCT CTC AGA GTT 3′ and 5′TGC CGC AGG CCT TTA GGA GCC AGG GAA ATA AAG 3′ containing the restriction sites Kpn1 and Stu1 was used to amplify 708 bp cDNA fragment of *AtSPL6*. The primer pair 5′GGC CTC GGT ACC GTT TTA TTC TTT CTC CTC TCA 3′ and 5′CGC TCC GAG CTC TTA GGA GCC AGG GAA ATA AAG 3′ containing restrictions sites for Kpn1 and Sac1 was used to amplify a 908 bp genomic fragment of *AtSPL6*. These PCR products were cloned into pYL400 vector in an anti-sense orientation to each other and downstream of a constitutive 35S promoter. The orientation of the two inserts was such that when transcribed, it would result in an RNA transcript with a double hairpin loop and stem structure. GV3101 Agrobacterium-containing *AtSPL6*-RNAi was transformed into Col-0 via the floral dip method [50]. Transformants were selected on Gentamycin (100 µg/mL) containing MS plates. Total RNA was isolated from 4-week old Col-0 and *AtSPL6*-RNAi plants using RNeasy Mini kit (QIAGEN). 3 µg of RNA was used for the synthesis of cDNA using SuperScript II reverse transcriptase (Invitrogen). Semi quantitative PCR was performed as described previously [27] using *AtSPL6* and *EF1α* specific primers. Two independent lines (#3 and #9) that showed significant downregulation of *AtSPL6* transcript were chosen for pathogen assays. Line #9 was used for microarray analysis.

### Quantitative real time PCR analysis of VIGS plants

Total RNA from VIGS plants was extracted using Plant RNeasy mini kit (QIAGEN). First strand cDNA was prepared from 1 µg total RNA using SuperScript II reverse transcriptase (Invitrogen). qPCR was performed using SYBR green (Applied Biosystems) in the ABI 7900 qPCR machine (Applied Biosystems). The fold change in mRNA levels was determined using the comparative Ct method after the data was normalized using EF1α as an internal control.

### *Pseudomonas* growth assays

*Pst*::avrRpm1, *Pst*::avrRpt2 and *Pst*::avrRps4 were grown on KM plates with appropriate antibiotics. The cells were harvested between 40–46 hrs; resuspended in 10 mM MgCl_2_, adjusted to 1×10^4^ cfu/mL and infiltrated onto 6 to 8 four-week old Col-0 and *AtSPL6*-RNAi plants. *Pst* DC3000 was infiltrated at a concentration of 1×10^6^ cfu/mL. Three leaves per plant were infiltrated for each line. Leaves of comparable age and at similar positions on the shoot were used for bacterial infiltration. The trays were covered with a humidity dome during the duration of the experiment. Bacterial growth curves were determined as described [51]. Each experiment was repeated three times.

### Microarray and quantitative real time PCR analysis

12 plants from 4-week old Col-0 and *AtSPL6*-RNAi were mock-infiltrated with 10 mM MgCl_2_ or with a high titer (1×10^7^ CFU/ml) of *Pst*::avrRps4. Total RNA from leaf samples harvested at 3 hpi and 6 hpi was extracted using Plant RNeasy mini kit (QIAGEN). cRNA preparation, hybridization and slide scanning was performed according to manufacturer's instructions (http://media.affymetrix.com/support/downloads/manuals/expression_analysis_technical_manual.pdf) at the WM. Keck Biotechnology Resource Laboratory, Yale University. A single array was run for the analysis. Gene expression intensities were calculated using the GC-RMA software [52] and normalized between slides via quartile normalization. Fold change values were calculated from the resulting signal intensities.

For real time PCR, first strand cDNA was prepared from 1 µg total RNA isolated from *Pst*::avrRps4 infected Col-0 and *SPL6*-RNAi plants using SuperScript II reverse transcriptase (Invitrogen). qPCR was performed using the iQ SyBR Green Supermix (Bio-Rad) in the Bio-Rad iCycler iQ multicolor real-time PCR system. Primary data analysis was performed with Bio-Rad iCycler iQ software. Relative RNA levels were calculated using the 2ΔΔC_t_ method after normalizing to the internal control Ubiquitin [53].

## Supporting Information

Figure S1 **Comparison of NbSPL6, NbSPL6_Like_ and AtSPL6 amino acid sequences.** The amino acid sequence of NbSPL6 compared with NbSPL6_Like_ and AtSPL6. Alignment was performed with ClustalW; identical and similar residues highlighted with the BoxShade program (http://www.ch.embnet.org/software/BOX_form.html). The italicized letters denote nuclear localization sequence (NLS). The line drawn above the sequence indicates the SBP DNA binding domain.
(TIF)

Figure S2 **N co-immunoprecipitates with NbSPL6 only during an active immune response.** Co-immunoprecipitation of gN-6xMyc with rNbSPL6-HA in the presence of the N eliciting p50-U1 or non-eliciting p50-Ob. Western blot analysis confirmed expression of the input proteins: gN-6xMyc (panel 1), tCFP-p50-U1 (panel 2, lanes 1 and 3), p50-Ob-tCFP (panel 2, lane 2), rNbSPL6-HA (panel 3, lanes 1 and 2), and NLS-GUS-HA (panel 3, lane 3). Due to high expression, NLS-GUS-HA (panel 3, lane 3) was adjusted to 1/50^th^ the volume loaded in lanes 1 and 2. Panel 4 shows the immunoprecipitated HA-tagged proteins. Asterisks show the immunoprecipitated rNbSPL6-HA and the arrow shows immunoprecipitated NLS-GUS-HA. Due to high expression, the IPed NLS-GUS-HA (panel 4) was adjusted to 1/50^th^ the volume loaded in lanes 1 and 2. gN-6xMyc co-immunoprecipitated with rNbSPL6 only in the tissue expressing tCFP-p50-U1 (panel 5, lane 1) but not in the tissue expressing p50-Ob-tCFP (panel 5, lane 2). gN-6xMyc did not co-immunoprecipitate with NLS-GUS-HA in the presence of tCFP-p50-U1 (panel 5, lane 3). M indicates marker. Protein sizes marked on the left are in kD.
(TIF)

Figure S3 **NbSPL6 is required for N mediated resistance to TMV-U1.** **A.** N-containing transgenic *N. benthamiana* plants were agro-infiltrated with an empty VIGS vector (VIGS-Vector), VIGS vector designed to silence *N* (VIGS-*N*) or *NbSPL6* (VIGS-*NbSPL6*). After 12 days, the plants were infected with TMV-U1 and monitored for the induction of the defense response. N-silenced plants and *NbSPL6*-silenced plants (middle and right panels) were unable to restrict TMV-U1 and the virus spread to the systemic un-inoculated leaves. This is characterized by trailing necrosis and collapse of the shoot (middle and right panels). The VIGS-Vector plants (left panels) could evoke complete resistance against TMV-U1. The bottom panels are enlarged images of the systemic, un-inoculated leaves from each plant. **B.** TMV-U1 inoculated leaves of VIGS-vector (left panel), N-silenced (middle panel) and NbSPL6-silenced plants (right panel). In the leaf from the control plant, the virus is restricted to the sites of inoculation (left panel). In the N and NbSPL6 silenced leaves, the virus escapes from the site of inoculation leading to its collapse (middle and right panel). **C.** The TMV coat protein (CP) transcripts were not detected in the upper un-inoculated tissue obtained from VIGS-Vector plants (top left panel but were detected in VIGS-*N* (top middle panel) and VIGS-*NbSPL6* plants (top right panel). NbEF1α was used as the internal control (bottom panels). Numbers above the gel indicate PCR cycles. M = DNA marker. **D.** Loss of N-mediated resistance to TMV. The number of plants that showed a loss of resistance to TMV is depicted. This was scored as plants showing accumulation of TMV in the upper uninoculated tissue and visible trailing HR-PCD/necrosis in the upper leaves.
(TIF)

Figure S4 ***AtSPL6*** **is required for RPS4-mediated defense against** ***Pst*** **::avrRps4 but not for basal resistance against** ***Pst*** **DC3000.** **A.** Semi-quantitative RT-PCR showing a significant reduction in *AtSPL6* transcripts in *AtSPL6*-RNAi plants line #3 (top panel, right) compared to Col-0 (top panel, left). *EF1α* was used as an internal control (bottom panel). Numbers above indicate PCR cycles. M = DNA marker. The semiquantitative RT-PCR data for transcript levels in *AtSPL6*-RNAi line #9 is shown in Figure 6. **B.** *Pst*::AvrRps4 growth in Col-0 (C), *AtSPL6*-RNAi line 3 (#3) and line 9 (#9), and *rps4-2* plants (r). *Pst*::AvrRps4 was syringe infiltrated and titers determined at 0 and 3 days post infiltration (dpi). Data from 2 biological replicates is shown. RPS4-mediated resistance to *Pst*::AvrRps4 is compromised in *AtSPL6*-RNAi plants and *rps4-2*. Student T test determined the difference to be statistically significant at α = 0.05 (*) and α = 0.01 (**). **C.** *Pst* DC3000 growth in Col-0 (C), *AtSPL6*-RNAi line 3 (#3) and line 9 (#9). *Pst* DC3000 was syringe infiltrated and titers determined at 0 and 3 dpi. Data from 2 biological replicates is shown. Statistical analysis revealed no significant difference in growth of *Pst* DC3000 between Col-0 and *At*SPL6-RNAi lines. Basal resistance against *Pst* DC3000 is not compromised in the two independent *AtSPL6*-RNAi lines. Experiments in B and C were done side-by-side with plants grown in the same growth trays and growth chamber.
(TIF)

Table S1 **Complete list of RPS4-induced genes that are downregulated 2 fold or more in SPL6-RNAi plants infected with Pst::avrRPS4 at 3 h or 6 h post infection.**
(PDF)

Table S2 **Primers used for Quantitative-PCR and Semi quantitative RT-PCR.**
(PDF)

Text S1 **Supplementary experimental procedures.**
(DOCX)
