# Supplementary figures and images for: Novel Positive Regulatory Role for the SPL6 Transcription Factor in the N TIR-NB-LRR Receptor-Mediated Plant Innate Immunity
Source: PLoS Pathog. 2013 Mar 14;9(3):e1003235. doi: 10.1371/journal.ppat.1003235 (PMC3597514; doi:10.1371/journal.ppat.1003235)

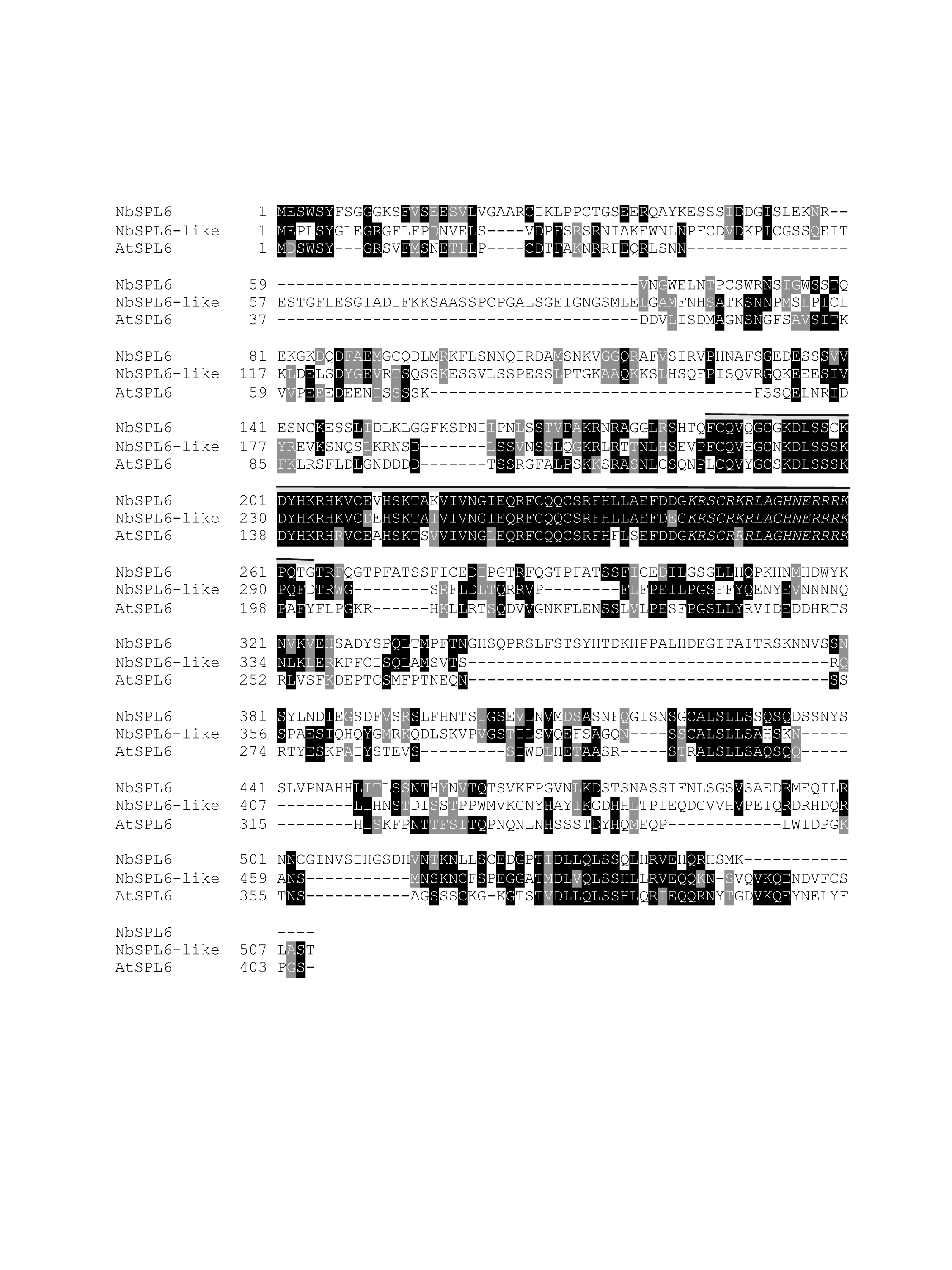

Supplement: Figure S1 — Comparison of NbSPL6, NbSPL6Like and AtSPL6 amino acid sequences. The amino acid sequence of NbSPL6 compared with NbSPL6Like and AtSPL6. Alignment was performed with ClustalW; identical and similar residues highlighted with the BoxShade program (http://www.ch.embnet.org/software/BOX_form.html). The italicized letters denote nuclear localization sequence (NLS). The line drawn above the sequence indicates the SBP DNA binding domain. (TIF) [file ppat.1003235.s001.tif]

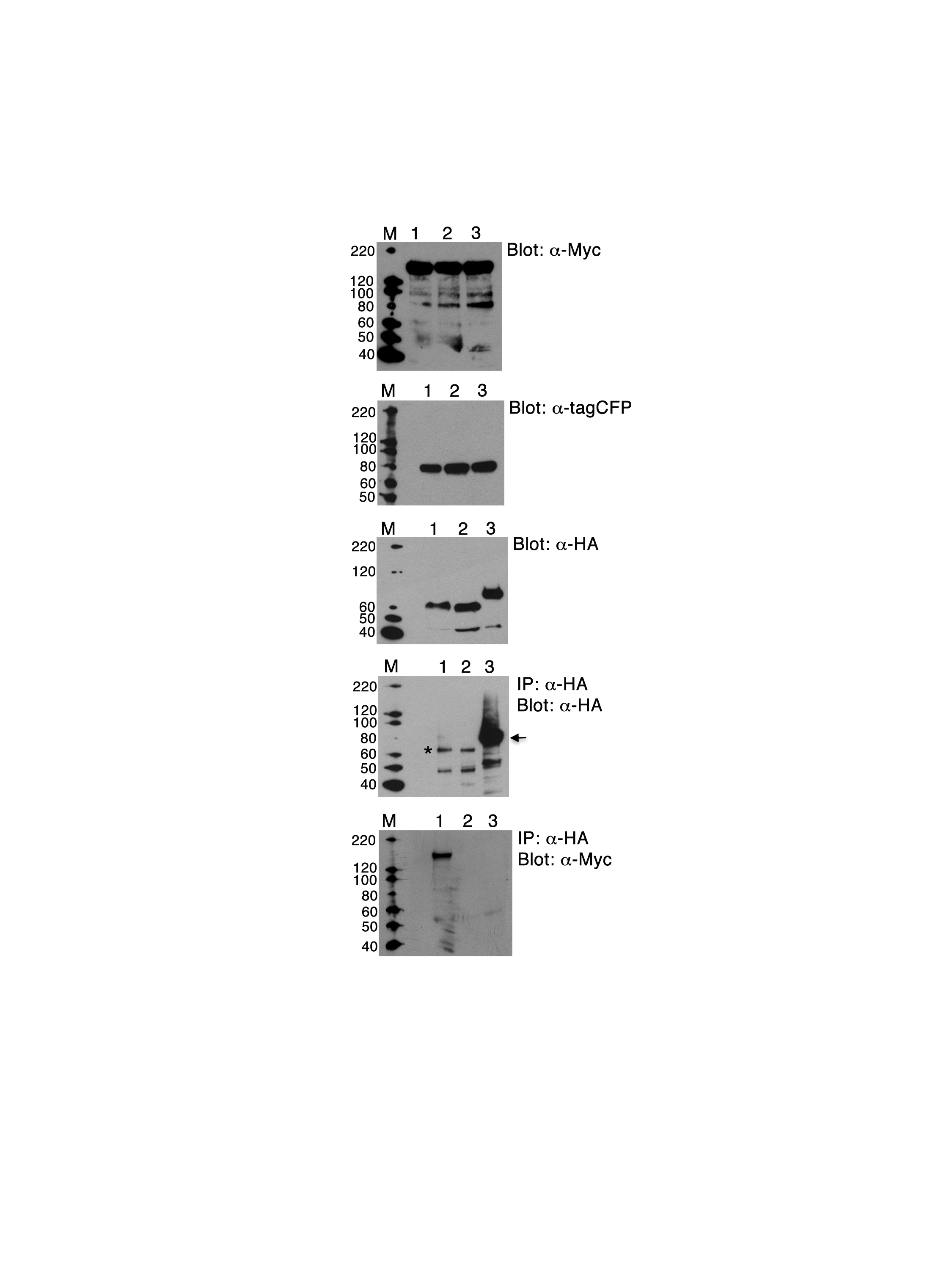

Supplement: Figure S2 — N co-immunoprecipitates with NbSPL6 only during an active immune response. Co-immunoprecipitation of gN-6xMyc with rNbSPL6-HA in the presence of the N eliciting p50-U1 or non-eliciting p50-Ob. Western blot analysis confirmed expression of the input proteins: gN-6xMyc (panel 1), tCFP-p50-U1 (panel 2, lanes 1 and 3), p50-Ob-tCFP (panel 2, lane 2), rNbSPL6-HA (panel 3, lanes 1 and 2), and NLS-GUS-HA (panel 3, lane 3). Due to high expression, NLS-GUS-HA (panel 3, lane 3) was adjusted to 1/50th the volume loaded in lanes 1 and 2. Panel 4 shows the immunoprecipitated HA-tagged proteins. Asterisks show the immunoprecipitated rNbSPL6-HA and the arrow shows immunoprecipitated NLS-GUS-HA. Due to high expression, the IPed NLS-GUS-HA (panel 4) was adjusted to 1/50th the volume loaded in lanes 1 and 2. gN-6xMyc co-immunoprecipitated with rNbSPL6 only in the tissue expressing tCFP-p50-U1 (panel 5, lane 1) but not in the tissue expressing p50-Ob-tCFP (panel 5, lane 2). gN-6xMyc did not co-immunoprecipitate with NLS-GUS-HA in the presence of tCFP-p50-U1 (panel 5, lane 3). M indicates marker. Protein sizes marked on the left are in kD. (TIF) [file ppat.1003235.s002.tif]

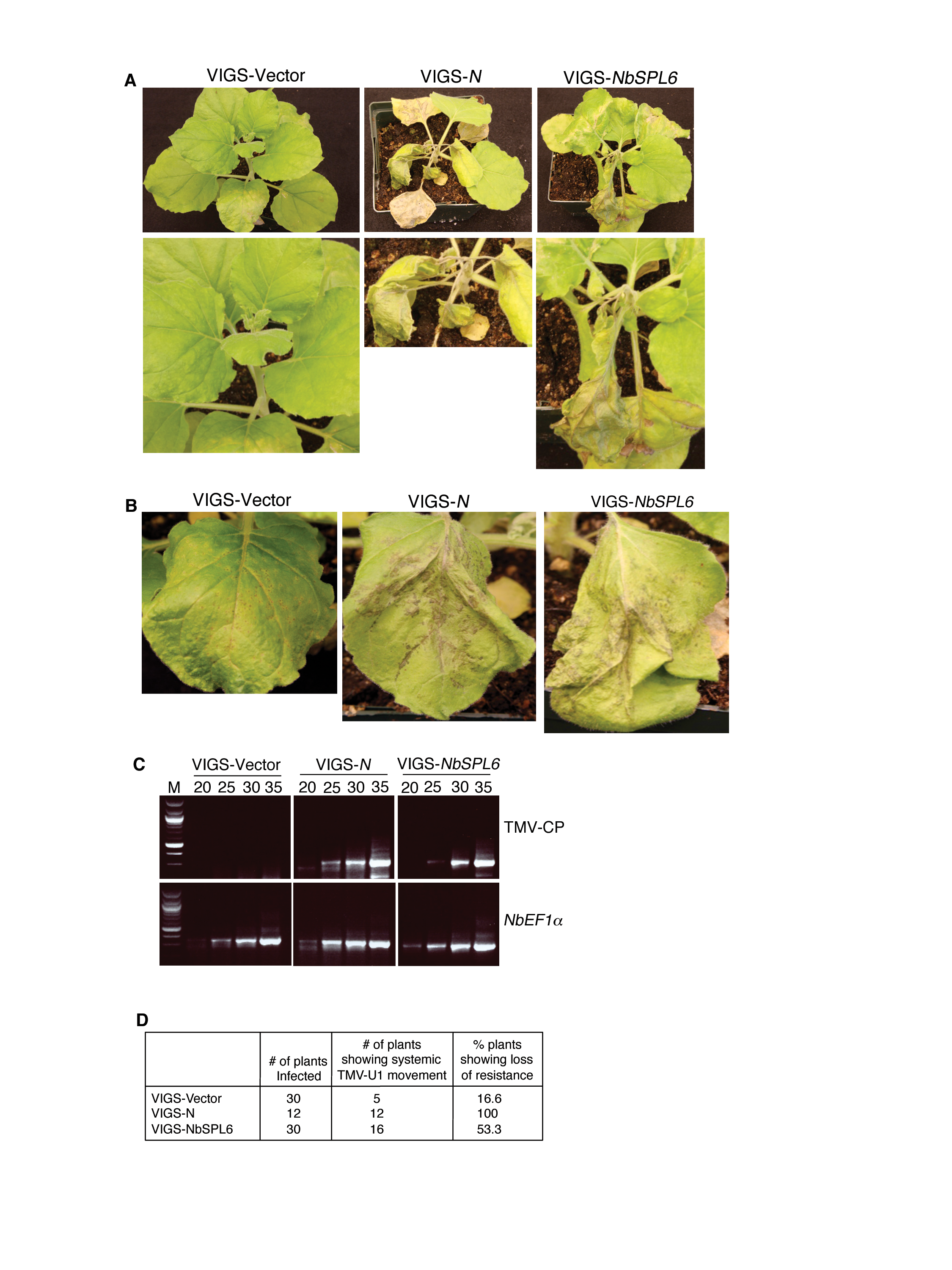

Supplement: Figure S3 — NbSPL6 is required for N mediated resistance to TMV-U1. A. N-containing transgenic N. benthamiana plants were agro-infiltrated with an empty VIGS vector (VIGS-Vector), VIGS vector designed to silence N (VIGS-N) or NbSPL6 (VIGS-NbSPL6). After 12 days, the plants were infected with TMV-U1 and monitored for the induction of the defense response. N-silenced plants and NbSPL6-silenced plants (middle and right panels) were unable to restrict TMV-U1 and the virus spread to the systemic un-inoculated leaves. This is characterized by trailing necrosis and collapse of the shoot (middle and right panels). The VIGS-Vector plants (left panels) could evoke complete resistance against TMV-U1. The bottom panels are enlarged images of the systemic, un-inoculated leaves from each plant. B. TMV-U1 inoculated leaves of VIGS-vector (left panel), N-silenced (middle panel) and NbSPL6-silenced plants (right panel). In the leaf from the control plant, the virus is restricted to the sites of inoculation (left panel). In the N and NbSPL6 silenced leaves, the virus escapes from the site of inoculation leading to its collapse (middle and right panel). C. The TMV coat protein (CP) transcripts were not detected in the upper un-inoculated tissue obtained from VIGS-Vector plants (top left panel but were detected in VIGS-N (top middle panel) and VIGS-NbSPL6 plants (top right panel). NbEF1α was used as the internal control (bottom panels). Numbers above the gel indicate PCR cycles. M = DNA marker. D. Loss of N-mediated resistance to TMV. The number of plants that showed a loss of resistance to TMV is depicted. This was scored as plants showing accumulation of TMV in the upper uninoculated tissue and visible trailing HR-PCD/necrosis in the upper leaves. (TIF) [file ppat.1003235.s003.tif]

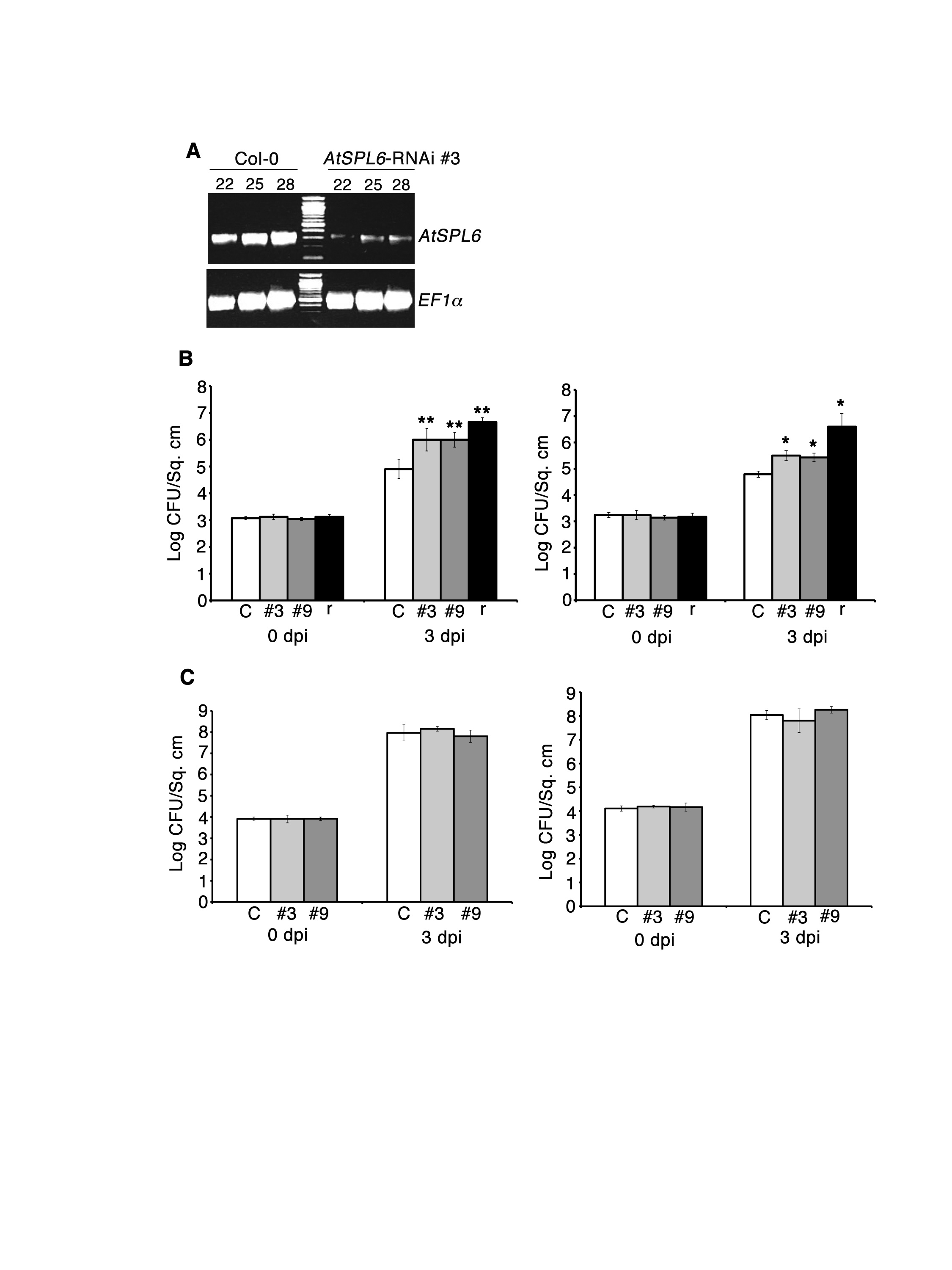

Supplement: Figure S4 — AtSPL6 is required for RPS4-mediated defense against Pst ::avrRps4 but not for basal resistance against Pst DC3000. A. Semi-quantitative RT-PCR showing a significant reduction in AtSPL6 transcripts in AtSPL6-RNAi plants line #3 (top panel, right) compared to Col-0 (top panel, left). EF1α was used as an internal control (bottom panel). Numbers above indicate PCR cycles. M = DNA marker. The semiquantitative RT-PCR data for transcript levels in AtSPL6-RNAi line #9 is shown in Figure 6. B. Pst::AvrRps4 growth in Col-0 (C), AtSPL6-RNAi line 3 (#3) and line 9 (#9), and rps4-2 plants (r). Pst::AvrRps4 was syringe infiltrated and titers determined at 0 and 3 days post infiltration (dpi). Data from 2 biological replicates is shown. RPS4-mediated resistance to Pst::AvrRps4 is compromised in AtSPL6-RNAi plants and rps4-2. Student T test determined the difference to be statistically significant at α = 0.05 (*) and α = 0.01 (**). C. Pst DC3000 growth in Col-0 (C), AtSPL6-RNAi line 3 (#3) and line 9 (#9). Pst DC3000 was syringe infiltrated and titers determined at 0 and 3 dpi. Data from 2 biological replicates is shown. Statistical analysis revealed no significant difference in growth of Pst DC3000 between Col-0 and AtSPL6-RNAi lines. Basal resistance against Pst DC3000 is not compromised in the two independent AtSPL6-RNAi lines. Experiments in B and C were done side-by-side with plants grown in the same growth trays and growth chamber. (TIF) [file ppat.1003235.s004.tif]
