# Supplementary material for: Novel Positive Regulatory Role for the SPL6 Transcription Factor in the N TIR-NB-LRR Receptor-Mediated Plant Innate Immunity
Source: PLoS Pathog. 2013 Mar 14;9(3):e1003235. doi: 10.1371/journal.ppat.1003235 (PMC3597514; doi:10.1371/journal.ppat.1003235)
Supplement: Table S1 — Complete list of RPS4-induced genes that are downregulated 2 fold or more in SPL6-RNAi plants infected with Pst::avrRPS4 at 3 h or 6 h post infection. (PDF) [file ppat.1003235.s005.pdf]

Table S1. Complete list of RPS4-induced genes that are downregulated 2 fold or more in SPL6-RNAi plants infected with Pst::avrRPS4 at 3 h or 6 h post infection

AT#	Annotation	log2 fold change SPL6-RNAi-infected (3h) vs. Col-0-infected (3h)	log2 fold change SPL6-RNAi-infected (6h) vs. Col-0-infected (6h)
At2g14560	unknown protein	-2.64	-1.25
At5g03350	putative protein	-2.54	-1.16
At4g17030	allergen like protein	-2.51	-1.04
At1g21110	O-methyltransferase, putative	-2.36	-0.47
At5g52760	putative protein	-2.33	-0.93
At2g37330	unknown protein	-2.14	0.36
At2g20145	Expressed protein	-2.07	-0.81
At5g41040	N-hydroxycinnamoyl/benzoyltransferase-like protein	-2.06	-0.22
At3g52430	putative protein	-2.05	-1.17
At1g73800	Expressed protein	-2.03	-0.83
At5g53030	putative protein	-2.02	0.24
At3g22620	hypothetical protein	-2.02	0.41
At2g14610	pathogenesis-related PR-1-like protein	-1.97	-3.46
At5g39670	calcium-binding protein - like	-1.96	-0.44
At3g52720	carbonic anhydrase (CAH1)	-1.90	0.39
At5g55450	unknown protein	-1.90	-2.40
At3g56400	DNA-binding protein 4 WRKY4 - Nicotiana tabacum	-1.88	-0.97
At3g60420	putative protein prib5	-1.86	-0.70
At5g47130	putative protein contains similarity to Bax inhibitor-1	-1.84	-1.62
At5g51190	putative protein contains similarity to ethylene responsive element binding factor	-1.80	0.20
At1g43910	unknown protein	-1.80	-0.70
At5g52750	putative protein	-1.78	-0.34
At5g54610	ankyrin-repeat-containing protein-like	-1.76	-1.15

At2g04450	putative mutT domain protein	-1.76	-2.92
At2g17040	NAM (no apical meristem)-like protein	-1.74	-0.61
At3g22600	unknown protein; lipid transfer protein family	-1.73	-0.43
At5g41610	Na+/H+ antiporter-like protein	-1.68	-0.91
At3g26470	unknown protein	-1.68	-1.08
At1g17610	disease resistance protein, putative	-1.67	-1.06
At1g35210	hypothetical protein	-1.63	-0.79
At5g15800	MADS box protein AGL2	-1.61	0.16
At1g33720	cytochrome P450, putative	-1.60	-0.92
At1g13470	hypothetical protein	-1.59	-1.73
At1g73805	putative calmodulin-binding protein	-1.59	-0.76
At1g18570	myb factor, putative	-1.56	-0.46
At1g72930	similar to flax rust resistance protein	-1.45	-0.96
At1g72240	hypothetical protein	-1.42	-0.38
At4g12690	putative protein	-1.41	-0.32
At3g50930	BCS1 protein-like protein	-1.40	-0.26
At4g33100	putative protein	-1.40	-0.96
At1g19960	hypothetical protein	-1.40	-0.83
At3g45640	mitogen-activated protein kinase 3	-1.39	-0.56
At4g17490	ethylene responsive element binding factor-like protein (AtERF6)	-1.39	0.38
At5g04070	putative protein	-1.38	-0.81
At3g20420	RNA helicase, putative	-1.37	-1.16
At5g58570	Expressed protein	-1.35	-0.48
At5g43910	putative protein	-1.30	-1.17
At5g05340	peroxidase	-1.29	0.27
At1g74710	isochorismate synthase (icsI)	-1.29	-0.81
At5g15860	Carboxylesterase-like	-1.28	-0.27
At2g31980	putative cysteine proteinase inhibitor B	-1.27	-0.02

At1g72920	virus resistance protein, putative	-1.26	-0.77
At3g22840	early light-induced protein	-1.24	0.60
At5g10380	putative protein	-1.23	-0.84
At3g09020	unknown protein	-1.23	-0.33
At2g26190	unknown protein	-1.23	-0.47
At4g14365	Expressed protein	-1.23	-0.81
At4g38560	Phospholipase like protein	-1.23	-1.54
At4g12720	growth factor like protein	-1.22	-0.42
At1g56520	disease resistance protein, putative	-1.21	-1.21
At1g72940	disease resistance protein, putative	-1.20	-0.66
At3g22231	Expressed protein	-1.20	-1.00
At4g04490	putative receptor-like protein kinase	-1.18	-1.49
At4g27280	putative protein centrin,	-1.17	-0.15
At4g26140	putative beta-galactosidase	-1.15	-0.82
At5g66380	putative protein	-1.15	-0.11
At1g68110	hypothetical protein	-1.15	-0.82
At2g42890	putative RNA-binding protein	-1.15	-0.72
At5g09470	mitochondrial carrier-like protein	-1.15	-0.55
At5g24110	unknown protein	-1.14	-0.15
At5g05460	similarity to endo-beta-N-acetylglucosaminidase	-1.14	-1.51
At1g60040	hypothetical protein	-1.14	1.05
At3g21080	unknown protein	-1.14	-0.90
At1g62710	beta-VPE	-1.14	-0.43
At3g04630	unknown protein	-1.12	0.56
At1g76620	unknown protein	-1.12	-1.68
At1g15790	unknown protein	-1.11	-0.85
At4g05460	F-box protein family, AtFBL20	-1.11	-0.17
At1g35230	hypothetical protein	-1.10	-1.07
At2g40750	hypothetical protein	-1.10	-0.20

At1g77760	nitrate reductase 1 (NR1)	-1.10	-0.74
At4g26550	putative protein	-1.08	-0.56
At5g60800	putative protein	-1.08	-0.97
At1g43790	hypothetical protein	-1.08	-0.68
At5g06480	putative protein	-1.07	-0.01
At1g74440	hypothetical protein	-1.06	-0.92
At2g32030	putative alanine acetyl transferase	-1.05	0.48
At1g33960	AIG1	-1.04	-0.87
At5g61900	copine - like protein copine I	-1.04	-0.88
At1g24140	putative metalloproteinase	-1.04	-0.12
At1g73830	putative helix-loop-helix DNA-binding protein	-1.04	-0.34
At3g47480	putative calcium-binding protein	-1.04	-0.91
At3g48080	hypothetical protein	-1.03	-1.22
At5g65205	Expressed protein	-1.02	-0.33
At1g72890	disease resistance protein, putative	-1.02	-0.73
At3g17365	Expressed protein	-1.01	-1.22
At2g13810	putative aspartate aminotransferase	-0.01	-4.77
At2g14620	putative endoxyloglucan glycosyltransferase	-0.03	-4.34
At1g21240	hypothetical protein	-0.02	-3.03
At1g33950	AIG1-like protein	-0.34	-2.92
At3g25010	disease resistance protein, putative	0.01	-2.87
At3g60470	putative protein	-0.01	-2.86
At5g11920	fructosidase - like	-0.65	-2.79
At3g12230	serine carboxypeptidase, putative	-0.01	-2.70
At5g45080	putative protein	0.01	-2.67
At3g12220	serine carboxypeptidase, putative	-0.03	-2.61
At5g55420	unknown protein	-0.01	-2.53
At1g35910	trehalose-phosphatase, putative	-0.02	-2.53
At5g26696	nectarin - like protein	1.04	-2.53

At3g13610	unknown protein contains similarity to DNA-binding protein	-0.01	-2.53
At1g04800	unknown protein	-0.22	-2.34
At2g23170	unknown protein	1.27	-2.33
At4g04500	putative receptor-like protein kinase	-0.02	-2.31
At2g18660	hypothetical protein	0.07	-2.24
At3g28510	hypothetical protein	0.94	-2.17
At5g40010	putative protein BCS1	0.54	-2.16
At1g63340	unknown protein contains similarity to Flavin-containing monooxygenase	-0.01	-2.13
At2g39320	hypothetical protein	-0.04	-2.07
At5g25820	putative protein	-0.02	-2.05
At5g41280	putative protein	0.72	-2.03
At4g10500	putative Fe(II)/ascorbate oxidase SRG1 protein	0.00	-2.03
At4g26270	pyrophosphate-dependent phosphofructo-1-kinase	0.09	-2.02
At1g21740	putative bzip-like transcription factor	0.00	-2.01
At4g36700	globulin-like protein	0.00	-2.01
At5g42830	N-hydroxycinnamoyl benzoyltransferase-like protein	0.32	-1.96
At1g17590	transcription factor, putative	-0.06	-1.95
At1g05880	hypothetical protein	-0.04	-1.94
At2g24650	hypothetical protein	-0.03	-1.94
At3g03700	hypothetical protein	-0.14	-1.93
At2g04430	putative mutT domain protein	-0.33	-1.92
At5g53870	putative protein	0.11	-1.91
At1g59500	auxin-regulated protein GH3, putative	0.33	-1.91
At1g17615	disease resistance protein RPP1-WsA, putative	0.00	-1.91
At4g18430	membrane-bound small GTP-binding - like protein	0.20	-1.90
At1g04490	unknown protein	-0.74	-1.90
At5g44460	calmodulin-like protein	0.05	-1.89
At1g21520	hypothetical protein	0.12	-1.87

At5g64780	putative protein	-0.06	-1.85
At5g52770	unknown protein	-0.09	-1.84
At5g22570	putative protein	0.22	-1.81
At1g03660	unknown unknown	0.04	-1.79
At1g19250	unknown protein similar to dimethylaniline monooxygenase	0.24	-1.78
At5g60280	receptor like protein kinase	0.23	-1.77
At3g59010	pectinesterase precursor-like protein	-0.11	-1.76
At5g45090	putative protein	-0.38	-1.74
At4g29020	glycine-rich protein like	1.05	-1.72
At4g23150	serine/threonine kinase - like	0.26	-1.70
At1g12290	hypothetical protein	-0.01	-1.70
At5g55170	ubiquitin-like protein	-0.84	-1.68
At3g51330	putative protein	0.01	-1.67
At3g57460	putative protein metalloendopeptidase	0.16	-1.67
At3g13090	ABC transporter, putative	1.37	-1.65
At5g61250	putative protein heparanase	-0.40	-1.65
At3g29250	short-chain alcohol dehydrogenase, putative	0.00	-1.64
At2g46070	putative mitogen-activated protein kinase	-0.98	-1.63
At4g05030	hypothetical protein	-0.01	-1.63
At4g15190	hypothetical protein	0.00	-1.62
At5g24530	flavanone 3-hydroxylase-like protein	-0.94	-1.60
At5g64800	CLE21, putative CLAVATA3/ESR-Related 21 (CLE21)	-0.03	-1.60
At4g36710	SCARECROW-like protein	-0.23	-1.59
At4g16820	triacylglycerol lipase like protein	0.06	-1.59
At1g03050	putative protein destination factor Similar to clathrin assembly protein	0.00	-1.55
At2g21030	hypothetical protein	0.10	-1.53
At1g02450	unknown protein	-0.06	-1.53
At5g23320	farnesyl cysteine carboxyl methyltransferase-like	-0.04	-1.51

At1g13310	hypothetical protein	0.67	-1.51
At4g00700	putative phosphoribosylanthranilate transferase	-0.86	-1.49
At4g23030	putative protein	0.09	-1.48
At5g41180	receptor kinase-like protein	-0.80	-1.48
At2g15040	putative disease resistance protein	-0.27	-1.45
At4g11840	putative phospholipase D-gamma	0.85	-1.45
At3g60520	putative protein	-0.73	-1.45
At4g11000	putative protein	-0.38	-1.45
At4g37010	caltractin-like protein	-0.61	-1.45
At3g50140	putative protein	0.70	-1.44
At4g12910	SERINE CARBOXYPEPTIDASE I PRECURSOR-like	-0.11	-1.44
At5g44480	putative protein	1.38	-1.43
At3g13782	nucleosome assembly protein, putative	0.27	-1.43
At5g51630	putative disease resistance protein; similarity to TMV resistance protein N	0.29	-1.42
At1g30900	putative vacuolar sorting receptor	0.00	-1.42
At5g01610	putative protein	0.04	-1.42
At1g74080	putative transcription factor	-0.03	-1.41
At2g26400	unknown protein	0.30	-1.41
At5g02690	putative protein	-0.52	-1.41
At5g57970	similarity to DNA-3-methyladenine glycosylase	-0.33	-1.41
At5g43150	unknown protein	-0.54	-1.41
At3g11340	glucosyl transferase, putative	0.63	-1.41
At4g39830	putative L-ascorbate oxidase L-ascorbate oxidase	-0.52	-1.41
At2g20110	hypothetical protein	0.09	-1.41
At3g26440	unknown protein	0.18	-1.40
At3g22930	calmodulin, putative	-0.77	-1.40
At3g26430	nodulin, putative	-0.70	-1.39
At5g09530	periaxin - like	-0.94	-1.39

At1g06770	hypothetical protein	0.27	-1.39
At2g43570	endochitinase isolog	0.30	-1.39
At1g67635	hypothetical protein	-0.02	-1.39
At1g17600	disease resistance protein RPP1-WsB, putative	-0.71	-1.39
At4g18630	putative protein	0.00	-1.39
At5g52810	putative protein contains similarity to ornithine cyclodeaminase	-0.11	-1.37
At3g54330	putative protein	-0.04	-1.37
At5g40730	putative protein	0.65	-1.37
At5g50560	putative protein	0.93	-1.36
At5g09290	3'(2'),5'-bisphosphate nucleotidase-like	-0.07	-1.36
At1g55610	receptor kinase, putative	0.10	-1.36
At5g18750	putative protein	0.58	-1.36
At3g04290	putative GDSL-motif lipase/acylhydrolase	2.09	-1.36
At5g11460	putative protein	-0.45	-1.34
At5g46280	MCM3 homolog	-0.03	-1.34
At2g02580	putative cytochrome P450	-0.20	-1.32
At5g45000	putative protein	0.68	-1.32
At5g13320	auxin-responsive - like	-0.62	-1.32
At5g40280	beta subunit of protein farnesyl transferase	-0.41	-1.31
At3g57240	beta-1,3-glucanase	-0.12	-1.30
At3g14470	disease resistance protein, putative	-0.57	-1.30
At1g76760	thioredoxin-like protein	0.33	-1.30
At5g58940	receptor-like protein kinase	-0.66	-1.30
At2g03410	unknown protein	0.88	-1.28
At2g16440	putative CDC21 protein	0.01	-1.27
At5g56970	cytokinin oxidase	0.62	-1.27
At3g51750	hypothetical protein	-0.91	-1.27
At4g30900	hypothetical protein	0.14	-1.26
At5g39190	germin-like protein (GLP2a)	-0.34	-1.25

At3g61390	putative protein	0.46	-1.25
At1g55780	hypothetical protein	0.00	-1.23
At5g01970	putative protein	0.01	-1.22
At3g60110	putative protein	0.10	-1.22
At4g26120	NPR1 like protein	-0.97	-1.22
At4g15200	p140mDia like protein	0.00	-1.22
At5g51380	putative protein	-0.38	-1.22
At1g51640	hypothetical protein	-0.14	-1.22
At2g25000	putative WRKY-type DNA binding protein	-0.91	-1.21
At1g33800	hypothetical protein	0.00	-1.21
At1g14870	unknown protein	0.63	-1.21
At1g10340	hypothetical protein	-0.81	-1.19
At1g21140	tonoplast intrinsic protein, alpha (alpha-TIP)	0.24	-1.19
At3g02770	putative S-adenosylmethionine:2-demethylmenaquinone	-0.68	-1.19
At1g07000	leucine zipper protein, putative	-0.15	-1.19
At5g26731	Expressed protein	0.00	-1.19
At3g26830	putative cytochrome P450	1.89	-1.19
At4g14630	germin precursor oxalate oxidase	0.02	-1.18
At1g02740	unknown protein	-0.30	-1.18
At2g31990	hypothetical protein	0.21	-1.18
At3g28580	hypothetical protein	-0.80	-1.17
At4g00234	predicted protein	0.01	-1.17
At4g32870	putative protein	0.81	-1.16
At4g33925	Expressed protein	-0.04	-1.16
At5g19580	putative protein glyoxal oxidase precursor	-0.86	-1.16
At5g17760	BCS1 - like protein	-0.09	-1.15
At3g16700	putative decarboxilase	-0.02	-1.15
At2g34500	putative cytochrome P450	1.83	-1.15
At1g61490	receptor kinase, putative	0.44	-1.15

At2g16870	putative disease resistance protein	-0.04	-1.15
At3g11040	unknown protein	0.00	-1.14
At3g15390	hypothetical protein	-0.02	-1.14
At3g48490	hypothetical protein	0.60	-1.14
At5g52720	putative protein	-0.79	-1.13
At5g64530	similarity to NAM (no apical meristem)	-0.58	-1.13
At3g52480	putative protein	-0.20	-1.12
At5g15870	putative protein beta-glucan-elicitor receptor	-0.39	-1.12
At1g24090	unknown protein	-0.17	-1.12
At1g65610	endo-1,4-beta-glucanase, putative	0.32	-1.12
At5g62770	putative protein	-0.07	-1.11
At3g57950	putative protein	-0.02	-1.11
At1g03055	Expressed protein	0.00	-1.11
At2g16835	putative plasma membrane intrinsic protein	0.38	-1.11
At1g01340	cyclic nucleotide and calmodulin-regulated ion channel, putative	-0.09	-1.09
At5g16310	ubiquitin C-terminal hydrolase-like	-0.54	-1.09
At4g00850	coded for by A. thaliana cDNA	-0.42	-1.09
At4g08110	predicted protein of unknown function	-0.40	-1.09
At3g45260	zinc finger protein	-0.90	-1.09
At2g26440	putative pectinesterase	-0.50	-1.09
At5g56340	putative protein	0.06	-1.08
At1g08180	hypothetical protein	-0.62	-1.08
At1g69526	Expressed protein	-0.57	-1.07
At5g11870	putative protein	0.64	-1.07
At4g16850	hypothetical protein	0.00	-1.07
At3g57100	putative protein	0.00	-1.07
At1g78550	flavanone 3-hydroxylase, putative	-0.04	-1.07
At2g40550	hypothetical protein	0.68	-1.06

At5g46230	unknown protein	-0.18	-1.06
At2g46000	unknown protein	-0.02	-1.06
At1g72730	putative Eukaryotic initiation factor 4A	-0.25	-1.06
At5g06750	protein phosphatase 2C-like	-0.26	-1.06
At4g11170	RPP1-WsA-like disease resistance protein	-0.06	-1.05
At2g22570	unknown protein	-0.01	-1.05
At1g31540	similar to downy mildew resistance protein RPP5 [Arabidopsis thaliana] GI:6449046	0.32	-1.05
At5g62180	putative protein PrMC3	0.30	-1.05
At4g21610	Lsd1 like protein zinc-finger protein	-0.04	-1.05
At4g19050	putative protein	-0.02	-1.05
At2g02930	putative glutathione S-transferase	0.26	-1.04
At1g03850	hypothetical protein	1.97	-1.04
At5g56610	putative protein	-0.18	-1.04
At3g17700	hypothetical protein	-0.51	-1.04
At5g45490	putative protein	-0.33	-1.04
At1g57650	disease resistance protein RPP1-WsA, putative	0.22	-1.04
At4g03450	hypothetical protein	-0.99	-1.04
At5g60930	microtubule-associated motor - like	0.04	-1.03
At2g29400	phosphoprotein phosphatase, type 1 catalytic subunit	-0.04	-1.03
At4g36550	putative protein	-0.62	-1.03
At3g53150	glucosyltransferase - like protein	0.14	-1.03
At2g16595	putative TRAP protein	-0.01	-1.03
At1g56540	disease resistance protein, putative	0.39	-1.03
At5g46520	disease resistance protein-like	-0.12	-1.03
At3g14060	expressed protein	-0.88	-1.02
At3g17420	serine/threonine protein kinase, putative	-0.06	-1.02
At1g61260	cotton fiber expressed protein, putative	-0.57	-1.02
At5g58980	random slug protein - like	0.26	-1.02

At4g23610	hypothetical protein	-0.31	-1.01
At1g75130	cytochrome P450, putative	-0.46	-1.01
At3g14700	hypothetical protein	0.54	-1.01
At3g61580	delta-8 sphingolipid desaturase	0.05	-1.01
At3g59470	putative protein	0.44	-1.01
At3g26840	unknown protein	-0.47	-1.01
At5g46960	unknown protein	0.00	-1.01
At3g48640	hypothetical protein	-0.86	-1.01
At2g44940	putative AP2 domain transcription factor	-0.13	-1.00
At4g02220	putative zinc finger protein	-0.29	-1.00
At5g55130	molybdopterin synthase sulphurylase	0.15	-1.00
At3g14840	receptor-like serine/threonine kinase, putative	-0.24	-1.00
At1g65870	dirigent protein, putative	-0.63	-1.00
At1g48320	hypothetical protein	-0.71	-1.00
At4g01680	putative transcription factor	0.40	-1.00
