# Supplementary material for: Novel Positive Regulatory Role for the SPL6 Transcription Factor in the N TIR-NB-LRR Receptor-Mediated Plant Innate Immunity
Source: PLoS Pathog. 2013 Mar 14;9(3):e1003235. doi: 10.1371/journal.ppat.1003235 (PMC3597514; doi:10.1371/journal.ppat.1003235)
Supplement: Table S2 — Primers used for Quantitative-PCR and Semi quantitative RT-PCR. (PDF) [file ppat.1003235.s006.pdf]

Table S2. Primers used for Quantitative-PCR and Semi quantitative RT-PCR

**Gene**	**Forward primer (5' – 3')**	**Reverse primer (5' – 3')**
NbSPL6	GCGAAGATATACTCGGCAGT	AAAGGCATGGTTAGTTGAGG
NbSPL6$_{like}$	ACCCTTTTTGTGATGTCGAT	GGACTACTGGCTGCTGATTT
NbEF1α	GGTCTACCAACCTTGACTGG	GACGTAGGGGTTTGTCTGAG
NbEF1α (sRT PCR)	GCTAGGTATGATGAAATCGTGAA	TCAACAGATTTAACTTCAGTTGT
AtNUDT5	CCTGCCAATGCGTCTCATC	TTGTCTTAATACCAGTTTCTTCTTCC
AtFMO1	TTGTCTTAATACCAGTTTCTTCTTCC	CTACGGCACGCAGAAGAG
AtPR1	CTTCCCTCGAAAGCTCAAGATAGCCCA	CCTCTTAGTTGTTCTGCGTAGCTCCGA
At2g20145	AAGTTTCAGGAGCAAAGACACTCGTG	GCTGGTAGTTCGTCATACAAGAAGG
At2g14620	GTACAGGCGAGTCTATGG	AAGCGTCTATCTTGTAATCC
AtALD1	TTCCATCGTATTGTGACCACTTCC	GTTGTTCACCGACCGTATCTCC
AtWAK3	TGTAATCATAATCGGCGTCTTGG	AACCCTGCTCCTGAAAGTCG
At3g28510	ATTGATTGCTCGCTTGATCTTACG	ACCTTGCTCTGCTTATCATCTACC
At3g22620	TTCGTGGTCAACAACAACCTCTAAGC	GAAGTTCCATTGCTGCTACTGTTGG
AtUBQ	TCACCGGAAAGACAATCACCCTCGAG	TCTAGCTGCTTGCCGGCGAAAATAAG
AtSPL6(sRT-PCR)	ATGGATTCTTGGAGCTACGGGAGAAG	AGATGGCAGAGCAAAACCTCTAGAG
AtEF1α (sRT-PCR)	GTGAGCACGCTCTTCTTGCT	TCCTTGACAGCAACATTATT
TMV repl. (sRT-PCR)	TTGTCATGAGCACTTCTTCGGTT	TTTCTCTAGATCTCTAATGATAC
