# Supplementary material for: Novel Positive Regulatory Role for the SPL6 Transcription Factor in the N TIR-NB-LRR Receptor-Mediated Plant Innate Immunity
Source: PLoS Pathog. 2013 Mar 14;9(3):e1003235. doi: 10.1371/journal.ppat.1003235 (PMC3597514; doi:10.1371/journal.ppat.1003235)
Supplement: Text S1 — Supplementary experimental procedures. (DOCX) [file ppat.1003235.s007.docx]

**Supplementary Experimental Procedures**

**Cloning NbSPL6**

Full-length *NbSPL6* was generated using the BD-SMART RACE cDNA Amplification kit (Clonetech). The primer pair 5’ATG GAA TCT TGG AGC TAC TTC TCT G3’ and 5’AGC ATC AAC GAC ATT CCA TGA AAT GA3’ was used for amplification.

**Plasmid construction**

The LexA-DNA binding domain containing bait plasmids LexA-TIR, LexA-NB, LexA-LRR, LexA-full-length N has been described previously [1,2]. PCR amplified NbSPL6, lacking the amino terminal 185 aa encoding the putative activation domain of NbSPL6, was cloned into pJG4-5 to generate the prey plasmid AD-NbSPL6. The plasmids gN-Yn, and p50-U1-HA have been described previously [1,3]. To generate gN^GK221-222AA^-Yn, XbaI-SphI fragment in gN-Yn was replaced with the PCR fragment containing the mutation. To generate p50-Ob-tCFP and p50-Ob-Yc, PCR amplified fragment containing nt2094-3413 from TMV-Ob [4] was cloned into tCFP vector SPDK1550 and Yc vector SPDK1103. To generate p126-U1-Cerulean and p126-Ob-tCFP, the p50 region was replaced with PCR amplified fragments of the 126 kD DNA fragments in p50-U1-Cerulean and p50-Ob-tCFP constructs. The PCR amplified full-length NbSPL6 and citrine was ligated into pYL400 (35S-MCS-NOS) to generate NbSPL6-Citrine. NbSPL6-HA was generated by PCR using a downstream primer containing 2xHA sequence. rNbSPL6 was generated by introducing 7 mutations into the predicted *miR156* binding site using PCR. To generate, rNbSPL6-HA, tagCFP in MSP78 (rNbSPL6-tagCFP) was replaced with the oligo containing the HA tag. To generate NbSPL6-Yc the PCR amplified NbSPL6 and Yc was ligated into pYL400. Inserting oligo containing HA tag between NbSPL6 and Yc in MSP51 generated NbSPL6-HA-Yc. TRV-NbSPL6 and TRV-NbSPL6_Like_ were generated by cloning the 313 bp and 300 bp regions spanning the 3’end of the coding sequence (CDS) and UTR region into pYL156 [5]. To generate NLS-GUS-HA, PCR amplified NLS-GUS and the HA oligo was cloned into pYL400. avrRps4-HA was generated by ligating PCR amplified avrRPS4 and the oligo containing HA tag into pYL400. The HA oligo was ligated between Yc and p50-U1 to generate Yc-HA-p50-U1. To generate p50-Ob-HA-Yc, the HA oligo was ligated between p50-Ob and Yc region. To generate gN-6xMyc, overlapping oligos containing 6xMyc was ligated into gN. To generate gN^GK221-222AA^-Myc, the XbaI-SphI fragment from gN^GK221-222AA^ was ligated into the gN-6xMyc construct cut with same enzymes. All constructs described above were confirmed by sequencing. All primers used for the construction of these plasmids are available upon request.

**Yeast two-hybrid assay**

The yeast two hybrid screening assay was performed as previously described [1,2]. Briefly, the yeast strain EGY48 containing the reporter plasmid pSH18-34 (with LEU2 as the reporter gene) and the bait plasmid LexA-LRR or LexA-N was transformed with a tomato cDNA library in the pJG-4-5 prey vector. Positive interactors were identified based on yeast growth on synthetic plates (SC) containing Galactose/Raffinose and lacking uracil, histidine, tryptophan and leucine after 2-3 days incubation at 30ºC. For the targeted yeast two-hybrid assays, pJG4-5 prey plasmid AD-NbSPL6, lacking the amino terminal activation domain of NbSPL6, was transformed into yeast EGY48/pSH18-34 with LexA-TIR, LexA-NB, LexA-LRR or LexA-full-length N bait plasmids. Positive interactors were determined as described above.

**RNA isolation and RT-PCR for detection of TMV RNA**

Systemic leaves of TRV2-Vector, TRV2-*N*, TRV2-*NbSPL6,* and TRV-*NbSPL6_Like_* silenced plants inoculated with TMV-U1 were used to isolate total RNA using the RNeasy Mini kit (QIAGEN). 2µg total RNA was used for the synthesis of first strand cDNA using SuperScriptII Reverse Transcriptase (Invitrogen). Semi quantitative RT-PCR was performed as previously described using gene specific primers against TMV replicase, TMV coat protein, and *N. benthamiana* *EF1α* [5].

**Supplemental References**

1. Caplan JL, Mamillapalli P, Burch-Smith TM, Czymmek K, Dinesh-Kumar SP (2008) Chloroplastic protein NRIP1 mediates innate immune receptor recognition of a viral effector. Cell 132: 449-462.

2. Liu Y, Schiff M, Serino G, Deng XW, Dinesh-Kumar SP (2002) Role of SCF ubiquitin-ligase and the COP9 signalosome in the N gene-mediated resistance response to Tobacco mosaic virus. Plant Cell 14: 1483-1496.

3. Burch-Smith TM, Schiff M, Caplan JL, Tsao J, Czymmek K, et al. (2007) A novel role for the TIR domain in association with pathogen-derived elicitors. PLoS Biol 5: e68.

4. Padgett HS, Beachy RN (1993) Analysis of a tobacco mosaic virus strain capable of overcoming N gene-mediated resistance. Plant Cell 5: 577-586.

5. Liu Y, Schiff M, Marathe R, Dinesh-Kumar SP (2002) Tobacco Rar1, EDS1 and NPR1/NIM1 like genes are required for N-mediated resistance to tobacco mosaic virus. Plant J 30: 415-429.
